# A Systematic Evaluation of Hospital Performance of Childbirth Delivery Modes and Associated Factors in the Friuli Venezia Giulia Region (North-Eastern Italy), 2005–2015

**DOI:** 10.1038/s41598-019-55389-z

**Published:** 2019-12-19

**Authors:** L. Cegolon, G. Mastrangelo, W. C. Heymann, G. Dal Pozzo, L. Ronfani, F. Barbone

**Affiliations:** 1 Local Health Unit N.2 “Marca Trevigiana”, Public Health Department, Treviso, Italy; 2 Institute for Maternal & Child Health, IRCCS “Burlo Garofolo”, Trieste, Italy; 3 Padua University, Department of Cardio-Thoracic & Vascular Sciences, Padua, Italy; 4 Florida State University, College of Medicine, Department of Clinical Sciences, Sarasota, Florida USA; 5 Florida Department of Health, Sarasota County Health Department, Sarasota, Florida USA; 6 Obstetrics & Gynecology Unit, Hospital “Villa Salus”, Venice, Italy

**Keywords:** Health policy, Health services, Epidemiology

## Abstract

Cesarean sections (CS) have become increasingly common in both developed and developing countries, raising legitimate concerns regarding their appropriateness. Since improvement of obstetric care at the hospital level needs quantitative evidence, using routinely collected health data we contrasted the performance of the 11 maternity centres (coded with an alphabetic letter A to K) of an Italian region, Friuli Venezia Giulia (FVG), during 2005–15, after removing the effect of several factors associated with different delivery modes (DM): spontaneous vaginal delivery (SVD), instrumental vaginal delivery (IVD), overall CS (OCS) and urgent/emergency CS (UCS). A multivariable logistic regression model was fitted for each individual DM, using a dichotomous outcome (1 = each DM; 0 = rest of hospital births) and comparing the stratum specific estimates of every term with their respective reference categories. Results were expressed as odds ratios (OR) with 95% confidence intervals (95%CI). The Benjamini-Hochberg (BH) false discovery rates (FDR) approach was applied to control alpha error due to the large number of statistical tests performed. In the entire FVG region during 2005–2015, SVD were 75,497 (69.1% out of all births), IVD were 7,281 (6.7%), OCS were 26,467 (24.2%) and UCS were 14,106 (12.9% of all births and 53.3% out of all CS). SVD were more likely (in descending order of statistical significance) with: higher number of previous livebirths; clerk/employed occupational status of the mother; gestational age <29 weeks; placentas weighing <500 g; stillbirth; premature rupture of membranes (PROM). IVD were predominantly more likely (in descending order of statistical significance) with: obstructed labour, non-reassuring fetal status, history of CS, labour analgesia, maternal age ≥35 and gestation >40 weeks. The principal factors associated with OCS were (in descending order of statistical significance): CS history, breech presentation, non-reassuring fetal status, obstructed labour, multiple birth, placental weight ≥ 600 g, eclampsia/pre-eclampsia, maternal age ≥ 35 and oligohydramnios. The most important risk factors for UCS were (in descending order of statistical significance): placenta previa/abruptio placenta/ antepartum hemorrage; non-reassuring fetal status, obstructed labour; breech presentation; PROM, eclampsia/pre-eclampsia; gestation 33–36 weeks; gestation 41+ weeks; oligohydramnios; birthweight <2,500 g, maternal age ≥ 35 and cord prolapse. After removing the effects of all other factors, we found great variability of DM rates across hospitals. Adjusting for all risk factors, all hospitals had a OCS risk higher than the referent (hospital G). Out of these 10 hospitals with increased adjusted risk of OCS, 9 (A, B, C, D, E, F, I, J, K) performed less SVD and 5 (A, C, D, I, J) less IVD. In the above 5 centres CS was therefore probably overused. The present study shows that routinely collected administrative data provide useful information for health planning and monitoring. Although the overall CS rate in FVG during 2005–15 was 24.2%, well below the corresponding average Italian national figure (38.1%), the variability of DM rates across FVG maternity centres could be targeted by policy interventions aimed at further reducing the recourse to unnecessary CS. The overuse of CS in nulliparas and repeat CS (RCS) should be carefully monitored and subject to audit.

## Introduction

Since childbirth is a natural event, all deliveries should ideally be spontaneous^1^. The recourse to instrumental vaginal deliveries (IVD) and cesarean sections (CS) should be made only if spontaneous vaginal deliveries (SVD) are not feasible, or to prevent maternal and perinatal mortality/morbidity^2–7^. When not medically justified, CS are not beneficial either for the woman or for the child, creating several types of health risks in both the short and long run, potentially stretching up for a long time after childbirth and also compromising future pregnancies, at higher risk of placenta previa, placenta accreta and gravid hysterectomy after a secondary CS^8–10^. CS is associated with obstetric complications ranging from maternal death, post-partum infection, uterine rupture, bladder injury, abnormal placentation, ectopic pregnancy, stillbirth, preterm birth, and others^2,9–12^. There is also growing evidence, albeit with different levels of strength, that CS modifies the flora of the bowel of the newborn and interferes with the development of the child’s immune system, therefore increasing the risk of allergies and obesity later in life^9^.

Furthermore, it is estimated that the average cost of a CS is at least two times higher than that of a SVD and may require the health care provider to perform additional and perhaps unnecessary obstetric procedures^13,14^. The average cost of a planned primary CS is reportedly 76% higher than the average cost of a SVD [$4,372 (95% CI: $4,293–4,451) vs. $2,487 (95%CI: $2,481–2,493)]^15^.

Nevertheless, CSs have become increasingly common almost everywhere, both in developed and developing countries, for a variety of reasons, raising legitimate concerns regarding their appropriateness^16–18^.

Given the above, experts appointed by the World Health Organization (WHO) back in 1985 issued international recommendations for CS rates (proportion of CS out of all deliveries) to not be higher than 10–15% in any region of the world^19,20^. Although the Nordic countries generally managed to meet or near the 15% CS threshold recommended by WHO^21^, starting from the 2009 edition of the *Monitoring Emergency Obstetric Care Handbook* of WHO the recommended CS rate of 15% has been questioned, given that”*there is no empirical evidence for an optimum percentage or range of percentages*”^22^. Some experts have even recently suggested that a cutoff of 19% for the CS rate could be more reasonable yet still effective in reducing maternal and neonatal morbidity/mortality^23^. Despite this change, a perception has remained that CS rates above such a “target” of 15% are unnecessary^22^.

To date, with only the exception of Africa, CS rates are higher than 15% and continuously increasing almost everywhere, both in high and low-income countries^2,24–26^. The highest global figures are in Latin America, where the average CS rates reach 40.5%^5^. The Dominican Republic is the country with the highest CS rate (58.1%) and South Sudan with the lowest (0.6%)^15^. The rates of CS are also considerably high in Turkey (47.9%) and Iran (47.5%)^5,27^. According to the most recent figures, Italy has the highest CS rate (38.1%) amongst all European countries, with high variability between the North (27.4%) and South (45.4%) of the country^5,28^. The overall annual cost attributable to CS in Italy is 103,505,894 USD, with an estimated 126,672 unnecessary CSs performed every year^29^.

Over the past two decades, the rise of CS in many countries has been offset by a corresponding decline in the IVD rates^30^. This decline seems influenced by the reluctance to perform IVD due to large accessibility and popularity of CS and the perceived health risks for the child associated with IVD. Since the rate of birth trauma increases with failed attempts at IVD, health care providers generally opt for a IVD only when its success is believed to be likely. However, the greatest issue with IVD is the difficulty in predicting its outcome, which is not the case with CS^31^.

A range of obstetric practices have become common over the past decades, despite being questionable from an evidence-based perspective^32^. Remarkable differences in DM rates are reported by geographical areas and individual maternity centres, a likely sign of low adherence to standardized protocols^33–35^. Quantifying and understanding hospital variability in the rates of DM is the starting point to plan health policies aimed at improving quality and efficiency of obstetric care^33^.

With this view, a prospective study was recently carried out in Friuli Venezia Giulia (FVG), a North-Eastern region of Italy, including all births occurring in its 11 maternity centres over a period of 18 months (July 2006-December 2007). In order to eliminate potential bias generated by different definitions and heterogeneity of data collection, a regional computerized database storing maternal characteristics, pregnancy-related factors, antenatal clinical risk factors, DM and short-term neonatal/maternal outcomes was developed ad hoc for the latter study and implemented prospectively in each maternity centre of FVG^36–38^.

Given the difficulties of repeating periodically similar studies to evaluate obstetric care services, we considered it important to conduct a further investigation on a larger scale and on a much longer timeframe, using administrative data routinely collected by the Italian National Health Service (NHS).

## Objectives

In view of the above, we carried out a population-based study in FVG during 2005–2015, to investigate patterns and factors associated with each different DM (SVD, IVD, overall CS (OCS) and urgent/emergency CS (UCS)), contrasting also the performance of the 11 maternity services of this Italian region.

## Methods

### Study design

As mentioned above, the present investigation employs a population-based cross-sectional design. Approval to conduct this study was granted by the Regional Health Authority of FVG.

### The database

Data from the 11 maternity services of FVG during calendar years 2005–2015 were extracted from the Regional Repository, a database anonymously storing administrative information from the Italian NHS. The database included information from two sources:

- hospital discharge forms (ICD-9 codes); and
- Certificate of Delivery Care (CEDAP, Italian acronym), which is a formatted questionnaire collecting clinical and personal information on women and newborns (Supplementary File)^39–41^.

We used the following ICD-9 codes to retrieve the obstetric conditions associated with each childbirth:

- Polyhydramnios: 657.0;
- Oligohydramnios: 658.0;
- Antepartum hemorrhage, abruptio placentae and placenta previa: 641.(0-1-2-3-8-9);
- Obstructed labour (except shoulder girdle dystocia): 660.(0-1-2-3-5-6-7-8-9);
- Non-reassuring fetal status: 656.3;
- Cord prolapse: 663.0;
- Premature rupture of membranes (PROM): 658.1;
- Eclampsia/pre-eclampsia: 642.(4-5-6-7);
- Rh iso-immunization: 656.1.

The rest of the data was collected from CEDAP. In particular, in our database DM was defined as follows:

1. Vaginal delivery (VD) without forceps or vacuum extraction;
2. Planned CS (PCS) or CS for failed induction;
3. CS during labour or urgent CS;
4. Forceps extraction;
5. Vacuum extraction;
6. Other forms of VD.

For the purpose of this study, we considered modalities 1 as SVD, modalities 4, 5 and 6 were combined in IVD, and categories 2 and 3 were incorporated into OCS. Category 3 indicates UCS.

The 12 regional facility centres were anonymized and coded by alphabetic letter from A to L. However, as stated above, maternity units of FVG were 11 (A to K). L is a major regional university hospital where only 12 complicated deliveries (7 PCS and 5 UCS) were referred during the entire study period (2005–2015). A and B are officially recognized second level maternity units (defined by the Italian Ministry of Health as facilities with >1,000 annual births and equipped with an neonatal intensive care unit), whereas the other 9 are first level (defined as facilities with <1,000 annual births and/or devoid of an neonatal intensive care unit).

**Figure 1:**
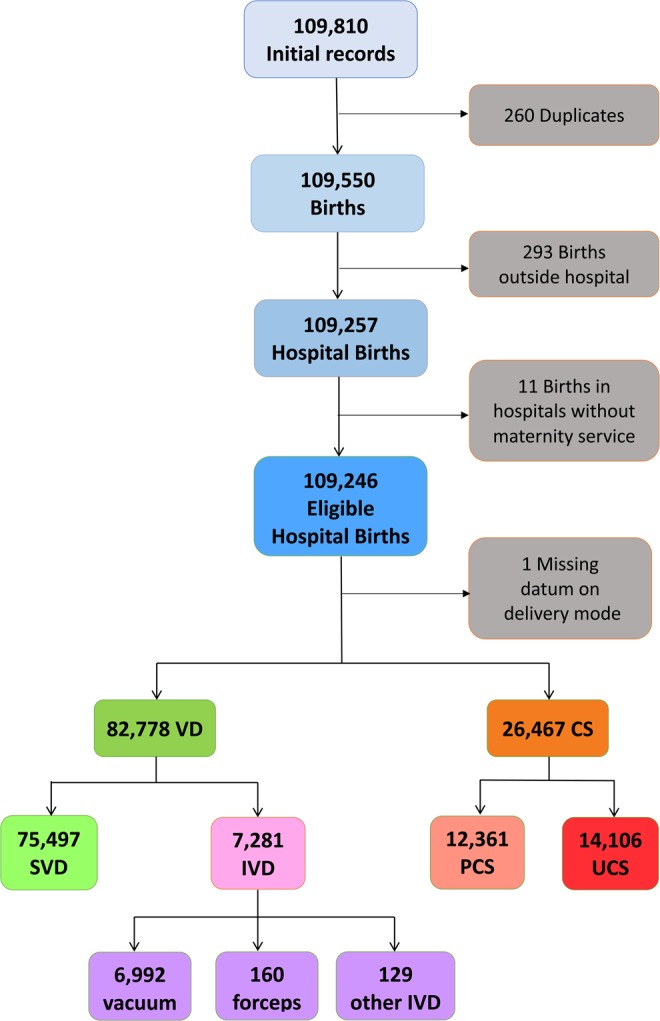
Flowchart displaying the criteria applied to the initial database to obtain the final number of hospital births available for the analysis. VD=Vaginal Deliveries; SVD = spontaneous vaginal deliveries; IVD = instrumental vaginal deliveries; CS = cesarean sections; PCS: Planned CS; UCS=Urgent/Emergency CS.

Figure 1 shows the flowchart displaying the various criteria applied to the initial database to obtain the final number of hospital records available for the analysis.

The rates of SVD, IVD, OCS and UCS in the entire FVG were calculated as percentages out of all births.

### Conceptual framework

**Figure 2:**
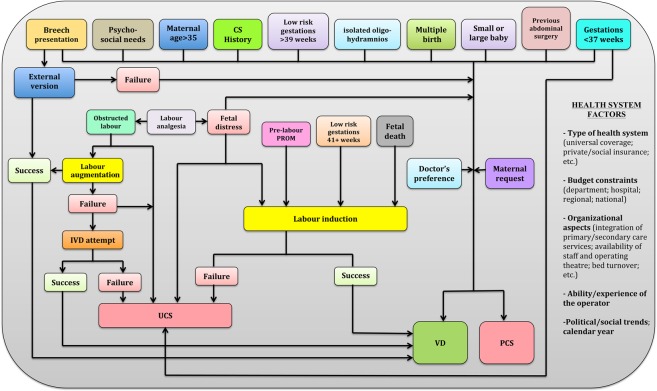
Conceptual framework (conceptualized by LC and GDP) explaining the relationships between the main determinants and the various delivery modes (DM). PROM = Premature Rupture of Membranes; VD = Vaginal Delivery; IVD = Instrumental Vaginal Delivery; CS = Cesarean Section; PCS = Planned CS; UCS = Urgent/Emergency CS.

Figure 2 displays the conceptual framework used to explain the relationship between the various factors (not all available in our analysis) involved and each DM. Four domains of potential determinants of DM were identified.

**Figure 3:**
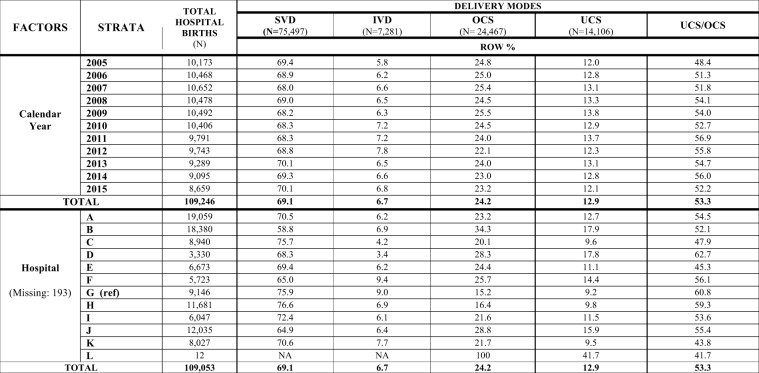
Distribution of delivery modes by calendar year and maternity centre. Number (N) and row percentage (row %); ref: reference category. SVD = Spontaneous Vaginal Deliveries; IVD = Instrumental Vaginal Deliveries; OCS = Overall Cesarean Sections; UCS = Urgent/Emergency Cesarean Sections. NA = Not applicable.

**Figure 4:**
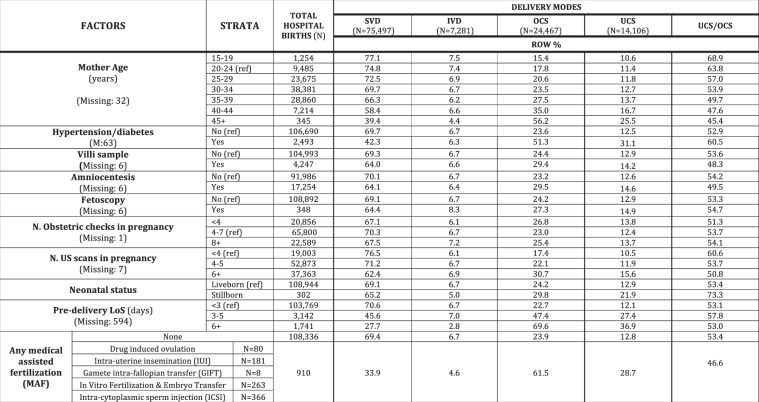
Distribution of delivery modes by maternal health factors. Number (N) and row percentage (row %); ref: reference category. SVD = Spontaneous Vaginal Deliveries; IVD = Instrumental Vaginal Deliveries; OCS = Overall Cesarean Sections; UCS = Urgent/Emergency Cesarean Sections.

**Figure 5:**
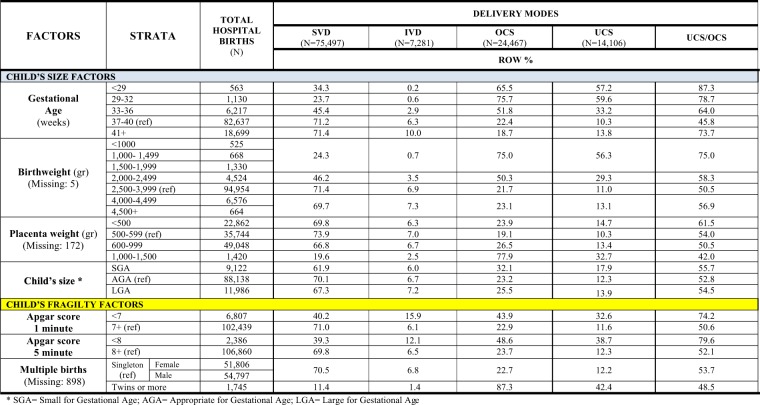
Distribution of delivery modes by clinical factors of the child. Number (N) and row percentage (row %); ref: reference category. SVD = Spontaneous Vaginal Deliveries; IVD = Instrumental Vaginal Deliveries; OCS = Overall Cesarean Sections; UCS = Urgent/Emergency Cesarean Sections.

**Figure 6:**
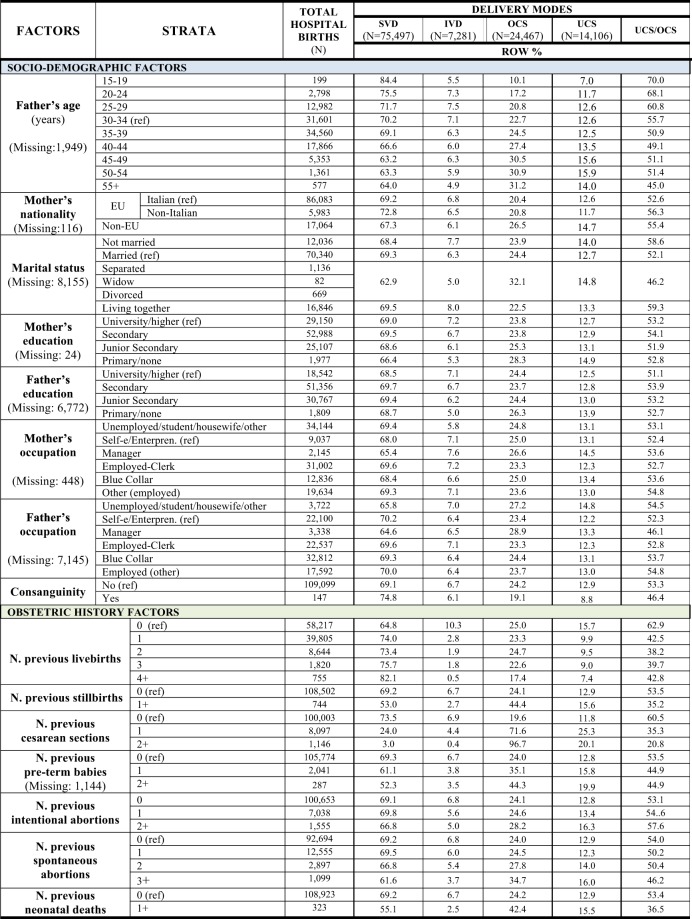
Distribution of delivery modes by socio-demographic and obstetric history factors. Number (N) and row percentage (row %); ref: reference category. SVD = Spontaneous Vaginal Deliveries; IVD = Instrumental Vaginal Deliveries; OCS = Overall Cesarean Sections; UCS = Urgent/Emergency Cesarean Sections.

**Figure 7:**
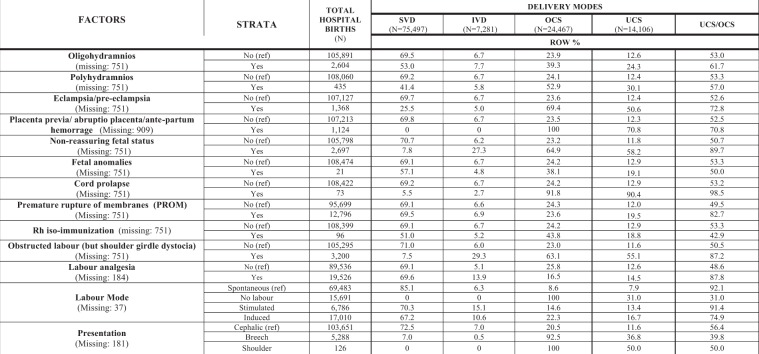
Distribution of delivery modes by obstetric factors. Number (N) and row percentage (row %); ref: reference category. SVD = Spontaneous Vaginal Deliveries; IVD = Instrumental Vaginal Deliveries; OCS = Overall Cesarean Sections; UCS = Urgent/Emergency Cesarean Sections.

1. Setting (hospitals) and timeframe (calendar year). The classes can be seen in Fig. 3.
2. Maternal health and child clinical factors. The classes are shown in Figs. 4 and 5
3. Socio-demographic background and obstetric history. The corresponding classes are displayed in Fig. 6.
4. Obstetric conditions, shown in Fig. 7: oligohydramnios; polyhydramnios; eclampsia/pre-eclampsia; placenta previa/abruptio placenta/ante-partum hemorrhage; non- reassuring fetal status; Congenital malformations at birth; cord prolapse; PROM; Rh immunization, obstructed labour (except shoulder girdle dystocia); labour analgesia; labour mode; presentation; any medical assisted fertilization.

### Data cleaning

As previously explained, some data check was conducted to clean potentially inconsistency of the information stored^39,40^. The following revisions have been made:

- 39 shoulder presentations delivering by SVD and 1 shoulder presentation delivering by IVD were reclassified as cephalic;

- 191 SVD as well as 48 IVD with no labour have been coded as missing;

- Placenta previa/abruptio placenta/ante-partum haemorrhage delivering by SVD (N = 136) and by IVD (N = 22) have been excluded from the analysis, for a total 158 missing data.

### Statistical analysis

We investigate the determinants of various DM (SVD, IVD, OCS, UCS) individually as compared to the rest of hospital births. Additionally we calculated the rate of UCS out of OCS (UCS/OCS).

We ran multiple logistic regression models using as outcome the variable 1/0 (1 = each DM vs. 0 = rest of hospital births). Since hospital G showed the best combination of optimal DM rates – the second highest rate of SVD (75.9%) and IVD (9.0%) as well as the lowest rate of OCS (15.2%) – it was chosen as reference facility in the statistical analysis.

Significant factors retained in each final logistic regression model were selected by backward stepwise procedure, setting the level of significance at 5%.

However, labour mode was dropped from all final multivariable models, because its stratum “*no labour*” comprised only PCS, thus generating collinearity issue between this variable and various DM outcomes at multivariable analysis.

Likewise, shoulder presentations (transverse fetal lies) were also excluded from all final multivariable models, since they were all delivered by CS.

Apgar score at 1 and 5 minutes were excluded since they were parameters measured after birth.

Furthermore, the following factors were dropped from the final multivariable logistic regression model, despite being significant at bivariate analysis adjusting only for hospital, since they were affected by a large number of missing values:

- **SVD**: marital status (p < 0.001); pre-term history (p < 0.001);
- **IVD:** marital status (p < 0.001); father’s education (p < 0.001); father’s occupation (p = 0.003); pre-term history (p < 0.001);
- **OCS**: marital status (p < 0.001); father’s education (p < 0.001); father’s occupation (p < 0.001); pre-term history (p < 0.001);
- **UCS**: marital status (p < 0.001); father’s education (p < 0.001); pre-term-history (p = 0.001);
- **UCS/OCS**: marital status (p < 0.001); father’s education (p = 0.005); father’s occupation (p = 0.005); pre-term-history (p = 0.001);

Considering the large number of statistical tests performed in all multiple logistic regression models, some p-values could have been significant by chance. Therefore, we employed a further selection procedure proposed by Benjamini-Hochberg (BH), setting the false discovery rate (FDR) at 5%^42^. Results were obtained by comparing each stratum specific estimate with the reference category and were expressed as odds ratios (OR) with 95% confidence interval (95%CI).

Given the percentage of missing values was less than 10% for all factors included in the multivariable logistic models, complete case analysis was adopted.

Stata 14.3 (College Station, Texas, USA) was employed for the analysis.

### Position statement

This work reports the scientific interpretation of obstetric data of FVG made by the authors, it should not be considered an official position of the Regional Government of FVG.

## Results

### Study subjects

Figure 1 shows the flowchart displaying the various selection criteria applied to the initial registered births (N = 109, 550) to obtain the final number of eligible hospital deliveries available for the analysis. In the whole FVG during 2005–2015 the total number of SVD were 75,497, IVD were 7,281 and OCS were 26,467. Among IVD, forceps were used in 160 cases; vacuum in 6,992 cases (96.0% of all IVD); the remaining assisted vaginal deliveries (VD) were 129 (1.8% of all IVD). Total PCS were 12,361 and UCS were 14,106.

### Descriptive analysis

**Figure 8:**
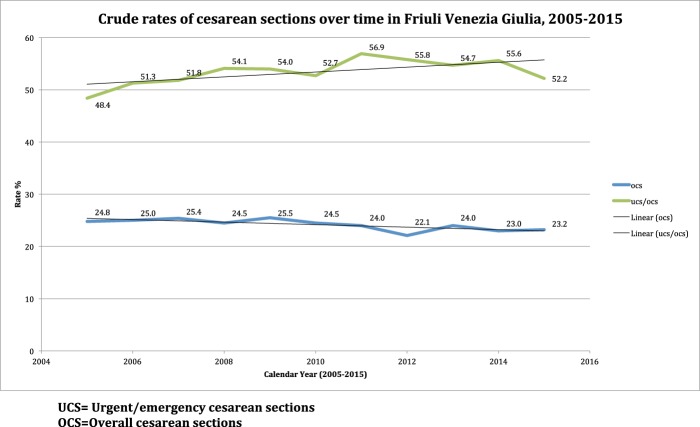
Crude rates of cesarean sections over time in Friuli Venezia Giulia during 2005–2015.

Figure 3 shows the distribution of DM by calendar year and maternity centre. In the entire FVG during 2005–2015 the rates of SVD, IVD and OCS were 69.1%, 6.7% and 24.2% respectively. UCS (CS during labour or urgent CS) were 12.9% of all births, 53.3% out of OCS. Whilst the crude rates of various DM were rather stable over time in the entire region, there was great variability across hospitals, particularly for OCS and UCS. Further, a slight decrease in the crude rate of OCS was accompanied by a relative increase of UCS/OCS over time (Fig. 8).

Figure 4 shows the distribution of crude rates of all DM by maternal health factors. Important factors for decreasing rates of SVD and higher rates of CS (especially UCS and UCS/OCS) were: (any) medical assisted fertilization; increasing maternal age; hypertension/diabetes; stillbirth and increasing pre-delivery length of hospital stay (LoS). IVD tended to decrease with maternal age and pre-delivery LoS.

Figure 5 displays the classes of clinical factors of the newborn. Inverse relationships between SVD and, on the other hand, IVD and CS were found for gestational age and multiple birth. The crude rates of OCS and UCS progressively decreased with gestational age, being higher for gestations <36 weeks. The proportion UCS/OCS was also high in gestations of 41+ weeks.

Figure 6 shows the classes of terms pertaining to the socio-demographic background and obstetric history. Although differences are minor, among socio-demographic factors higher crude rates of OCS were found for single women, non-EU nationality of the woman, increasing father’s age, lower maternal education and fathers that were occupied as managers. The latter figures were reversed for UCS, with exception of paternal age, although UCS/OCS diminished with father’s age.

As regards to obstetric history, 1 or ≥2 previous CS had the highest OCS rates, with lower respective figures for UCS and a decreasing pattern for UCS/OCS. Other important factors for the OCS rates were previous stillbirth, increasing number of previous pre-term babies, previous neonatal deaths and ≥2 previous spontaneous abortions. Among UCS, the highest proportions corresponded to ≥1 previous CS, previous stillbirths, ≥1 past pre-term babies, previous neonatal deaths and ≥3 spontaneous abortions.

Figure 7 displays the obstetric conditions related with childbirth. The highest crude rates of OCS and UCS were found for cord prolapse, placenta previa/abrupto placenta/antepartum hemorrage, shoulder as well as breech presentation, obstructed labour, non-reassuring fetal status and eclampsia/pre-eclapmsia. The IVD rate increased with obstructed labour, non-reassuring fetal status, labour analgesia and labour induction.

### Outcome results: the determinants

The results of Supplementary Tables 1 and 2 (Supplementary File) belong to the same multiple logistic regression models, one for each DM (SVD, IVD, OCS, UCS and UCS/OCS); they have been split in two tables to better view the effects of maternity centre adjusted for all other factors listed in the footnotes of both Supplementary Tables 1 and 2.

DM are reported in columns, significant factors in rows and the corresponding OR, 95%CI and BH p-value at each row/column interception. We focused only on positive associations, nevertheless negative associations are also displayed.

A can be seen from Supplementary Table 1 (Supplementary File), the main factors associated with increased probability of SVD (in descending order of BH p-value) were: higher number of previous livebirths; clerk/employed occupational status of the mother; gestational age <29 weeks; placenta weighing <500 g; stillbirth and PROM.

By contrast, IVD were principally associated with (in descending order of BH p-value): obstructed labour non-reassuring fetal status; labour analgesia; history of one previous CS; gestational age 41+ weeks; maternal age 40–44 years; non-EU nationality of the woman and increasing calendar year.

OCS were more likely with (in descending order of BH p-value): CS history; breech presentation; non-reassuring fetal status; obstructed labour; placental weight ≥600 g multiple births;; eclampsia/pre-eclampsia; maternal age 40–44 years; oligohydramnios; pre-delivery LoS ≥3 days; birthweight <2,000 g; maternal age ≥45 years; pre-term gestations (33–36 weeks); birthweight 2,000–2,500 g; 6+ US scans during pregnancy; polyhydramnios; cord prolapse; non-EU nationality of the woman; lower maternal education (<secondary); 4–5 US scans during pregnancy; hypertension/diabetes; Rh iso-immunization and 29–32 week gestations.

The most important factors associated with enhanced risk of UCS were (in descending order of BH p-value): placenta previa/abruptio placenta/antepartum hemorrage; non-reassuring fetal status, obstructed labour; CS hisory; breech presentation; PROM, eclampisa/pre-eclampsia; gestation 33–36 weeks; gestation 41+ weeks; oligohydramnios; birthweight <2,000 g, maternal age 40–44 years; birthweight 2,000–2,500 g; cord prolapse; placental weight (600–999) g; mother’s age 35–39 years; gestations 29–32 weeks; labour analgesia; polyhydramnios; mother’s age 30–34; non-EU nationality of the woman; multiple birth; mother’s age ≥45 years; gestations <29 weeks, placental weight 1,000–1,500 g and birthweight ≥4,000 g.

The adjusted risk of UCS over OCS (UCS/OCS) was higher with (in descending order of BH significance): PROM; labour analgesia; non reassuring fetal status; obstructed labour; pre-term (<36 weeks) and post term (41+ weeks) gestations; placenta previa/placenta accreta/abruptio placenta; eclampsia/pre-eclampsia; increasing calendar year; birthweight <2,500 g and non-EU nationality of the woman.

### Outcome results: the maternity centres

Supplementary Table 2 (Supplementary File) displays the maternity centres of FVG in the rows, the DM in the columns and the corresponding OR, 95%CI and BH p-value at the interception of rows and columns. As explained above, the risk estimates for each DM of Supplemetary Tables 1 and 2 are adjusted for the same factors displayed at the bottom of them.

All maternity centres presented higher OCS risks compare to the reference hospital (centre G). With the exception of hospital K (not differing from the reference) all hospitals were more likely to perform UCS. Facility F was the only maternity unit more likely to deliver IVD. All maternity units but H were far less likely to perform SVD as compared to the reference centre G.

## Discussion

### Strengths and limitations

Our study has several strengths. It is a population-based study with very high completeness and quality of data. In fact, our analysis took into account the effect of a considerable number of factors (including those recommended by the Robson classification system to compare CS rates) that may affect decisions-making on DM. Health data we analyzed were collected by trained health care staff. The large number of records available for the analysis allowed substantial statistical power and accuracy of results. The proportion of missing values was negligible, and mainly related to socio-demographic information. Lastly, we employed the BH procedure to control Type 1 errors due to multiple testing.

We had no direct information on the indication for each CS, including patient’s choice or doctor’s preference, factors increasingly influencing the decision to opt for a CS without medical indication^20^.

Other limitations are the lack of information on body mass index (BMI), Bishop index, smoking habits and physical exercise, risk factors for CS^43^. Furthermore, the labour mode variable, which includes information on labour induction, was dropped from all final multivariable models, because of collinearity. Finally, the use of the ICD-9 codes selected to retrieve the obstetric conditions associated with childbirth may be problematic, because the codes in some instances may be ambiguous in describing the associated conditions (i.e. the code 658.1, “Premature rupture of membranes”, does not distinguish between PROM and preterm rupture of membranes, which are two different conditions).

Although our regression models were adjusted also for birthweight and placental weight (parameters measured after birth), their causal effect on prenatal decision making on DM was excluded.

As mentioned above, the epidemiological patterns of CS and IVD were previously investigated in FVG through a comprehensive, prospective, ad hoc data collection, limited in time and too complex to be repeated periodically^36–38^. The present study shows that routinely collected administrative health data provide useful information for health planning and monitoring. They are relatively inexpensive, do not require ad hoc collections, are accessible, annually updated, allowing for the assessment of time trends. Furthermore, in addition of being the first international publication on childbirth DM using CEDAP data in FVG, compared to above mentioned previous studies conducted in FVG, the present work relies on a remarkably greater sample (110,950 vs.15,726 hospital births), on a much longer timeframe (11 vs. 1.5 years), examining all possible DM and controlling for a higher number of explanatory factors. To the best of our knowledge there are no other studies using a similar comprehensive and systematic methodology.

### Instrumental vaginal deliveries

The overall rate of IVD in FVG during 2005–2015 was 6.7%, higher than the recent Italian national picture (3.4%) and similar to reports from Germany (6.4%) and Austria (5.6%). However, recent figures from other Western European countries showed higher IVD rates, in particular Ireland (16.4%), France (12.1%), Belgium (10.9%), Luxembourg (10.2%), the Netherlands (10%), Switzerland (11.0%), England/Wales (10%), Norway (9.9%), Denmark (9.4%), Finland (8.6%) and Sweden (7.6%)^44^. Interestingly, Central-Eastern European countries showed substantially lower IVD rates, especially Slovenia (3.5%), the Czech Republic (1.8%), Latvia (1.6%), Lithuania (1.3%) and Romania (0.5%), confirming that obstetric practices in these countries are considerably different from Western Europe and are probably influenced by previous communist health policies still persisting nowadays^39,44^. IVD rates close to Western European countries are reported from other overseas high-income nations such as Australia (11%) and Canada (10.7%)^45^. Whilst in the UK the rates of IVD have remained relatively stable (10–13%) over the years, in North America it is considered a declining practice, especially in the USA^30,46,47^, where the IVD rate notably decreased from 9.4% in 1995 to 3.1% in 2017, and the CS rate considerably increased from 20.8% in 1995 up to 32.0% in 2017^46,48–50^. The decrease in the IVD rate was less prominent in Canada, but still decreased from 16.8% of all VD in 1995 to 13.2% in 2014, with the CS rate increasing from 17.6% up to 27.3%^46,51,52^. As mentioned in the introduction, declining IVD rates generally correspond to increasingly higher CS rates, and an increased use of IVD has been recommended as one of the strategic approaches to contain the recourse to unnecessary CS^46,47^. From this perspective, we observed a mild yet encouraging increase in the adjusted rate of IVD over the years in the entire FVG during 2005–2015.

IVD is employed to cut second stage labour for particular clinical conditions either of the fetus or the mother, balancing the risks and benefits of continued pushing versus an IVD^47,53^. In case of failed progressive descent of the fetal head at each vacuum pull or if child delivery is still not forthcoming after 3 correct pull attempts performed by an experienced operator, IVD should be abandoned in favour of a CS^50,53^. Under conditions that may increase the risk of IVD failure (especially maternal BMI >30, fetal macrosomia, occipito-posterior position and mid-cavity delivery) labour induction should be considered with the view of undertaking a UCS^47,53,54^.

Epidural analgesia increases the risk of IVD and labour induction by prolonging second stage labour by 15 to 30 minutes^55–58^. Non-reassuring fetal status during labour is reported in 10–20% cases following administration of epidural analgesia, although with no heath impact on the infant^59^. The association between history of one previous CS and IVD we found is interesting since Vaginal Birth After Cesarean Delivery (VBAC) is highly encouraged with the goal of reducing the number redundant CS, where feasible^30,60^.

The choice of the instrument to perform IVD should take into account the clinical conditions of the parturient and the experience/ability of the operator. Forceps and vacuum have different pros and cons. Although failed attempts of IVD are more likely with vacuum extraction, obstetricians and international training programs still appear to prefer it over forceps, since the latter requires considerable experience for its correct use^47,53^. In FVG during 2005–2015 vacuum was used in 96% of all IVD. In the USA during 2017 vacuum and forceps were respectively employed in 2.6% and 0.6% out of all VD, with wide geographical variation across and within the country^49,50^.

### Cesarean sections

The OCS rate in FVG during 2005–2015 was 24.2%. Despite this figure being higher than the threshold recommended by WHO and those achieved by the Nordic countries, it is well below the most recent figures reported for the entirety of Italy (38.1%), slightly lower the corresponding OCS rate in the north of the country (27.4%) and under the most recent average figures from Organization for Economic Cooperation and Development (OECD) countries (27.6%)^5,20,21^.

The recourse to CS has become pandemic worldwide, especially in Latin America, with significant differences between and within countries^5,18^. The underlying reasons for these increasingly high figures are certainly multiple^61^.

According to the open literature, the primary clinical conditions influencing a decision to perform a CS are obstructed labour, breech presentation, repeat CS (RCS) and non-reassuring fetal status^61–64^. An audit conducted in England and Wales ranked the proportions of CS by the above indications as follows: non-reassuring fetal status (22.0%); obstructed labour (20.4%); previous CS (13.8%); and breech presentation (10.8%)^64^. Among centres affiliated to the Consortium of Safe Labour from 2002–2008 in the USA, the most important indications to perform 38,484 primary CS were obstructed labour (35.4%), non-reassuring fetal status (27.3%) and fetal mal-presentation (18.5%)^65^.

The above were also the most prominent factors associated with both OCS and UCS at multivariable analysis in the present investigation, followed by eclampsia/pre-ecampsia and oligohydramnios. However, whilst the level of significance of the respective associations was relatively balanced between OCS and UCS for all latter conditions, the respective risk estimates for CS history and breech presentation were much stronger for OCS than UCS, hinting prevalent planned obstetric procedures.

A recent survey on 1,000 Italian women estimated that 20% respondents preferred to deliver by CS^66^, a proportion much higher than the pooled estimate reported for the entire Europe (11%)^67^. The main reasons a woman preferred a CS were perceived pain associated with VD, perceived higher safety of CS both for the mother and the newborn and the opportunity to plan childbirth^66^. Additionally, even in some non-obstetric clinical conditions (as for instance a history of abdominal surgery, including also bowel resection for endometriosis), CS may be perceived safer than VD and performed on maternal request or on doctor’s preference, without medical indication^68–71^. However, qualitative research highlighted that women are influenced predominantly by their obstetrician in decision-making on DM, especially if they already had a CS^66^. Whilst most women reportedly prefer SVD before having a CS, they then rate VD as less safe than CS following recommendations from their obstetrician^66^.

Obstructed labour and non-reassuring fetal status are both considered grey clinical areas potentially affected by issues of misclassification, ambiguity of diagnosis and practice pattern, questioning the real benefits of a CS for the patient^61,65,72^. Moreover, fetal heart rate monitoring for the detection of fetal asphyxia during labour is featured by low specificity^73^.

As regards to obstructed labour (highly inter-connected with labour induction) CS should be averted before the establishment of an active phase (employing a 6 cm benchmark for cervix dilation), especially in nulliparas and in trial of labour after CS^74^. Prediction models have recently been devised and validated both for nulliparas and multiparas undergoing obstructed labour, counseling women on the risk of CS based on some relevant parameters: parity; BMI; gestational age; presentation and Bishop index^43^.

To decrease the number of PCS international guidelines recommend vaginal breech extraction by external rotation of near term fetuses with breech presentation where appropriate^75^.

Multiple births and placental weight ≥600g (which may mask the effect of multiples) were the following relevant factors predominantly associated with OCS than UCS in our analysis. Twin pregnancies account for a small fraction of all births, hence encouraging VD in this group will have negligible impact on the reduction of the primary CS rate. However, this will have an impact in future pregnancies, at higher risk of RCS. In the whole FVG the percentage of twin pregnancies decreased from 1.9% (=191/10,087) in 2005 to 1.1% (=92/8,610) in 2015. By contrast, the proportion of breech presentations among multiples was rather constant over time, being 32.3% (=60/186) in 2005 and 34.8% (=32/92) in 2015. The rate of OCS among multiples in the whole region was high and rather stable over the years, being 90.1% (=172/191) in 2005, 88.7% (=194/219) in 2010 and 90.2% (=83/92) in 2015. Likewise, the rate of OCS among twin pregnancies with breech presentation was very high and relatively stable over time in the entire FVG, being 98.3% (=59/60) in 2005, 96.7% (=64/66) in 2010 and 100% (=32/32) in 2015. National data from the USA showed lower CS rates in twin pregnancies, although constantly increasingly from 53.4% in 1995 up to 75.0% in 2008, with a 5% yearly enhancement^76^. Whilst cephalic-cephalic twin pregnancies should all be considered for VD and a non-cephalic presentation of the leading twin is an indication for a CS, the evidence on the appropriate DM for cephalic/non-cephalic twins is inconclusive^77,78^. We did not have information on cephalic/non-cephalic twins in our study. The reported lack of dexterity required for a VD in case of malpresenting twins is pushing obstetricians to opt for a CS, contributing to decrease their confidence in vaginal breech extraction with cefalic/non-cefalic twins, therefore creating a vicious circle plumping up the CS rate^78^.

Eclampsia/pre-eclampsia and oligohydramnios were the following relevant factors, associated with rather balanced level of significance with both OCS and UCS. In case of eclampsia/pre-eclampsia, the severity of the condition may require labour induction before 39 week gestation^79,80^. As to oligohydramnios, labour was induced in 58.1% (=1,508/2,597) cases in the whole FVG during the study period. Although there is evidence it is a risk factor for labour induction, CS and infant morbidity, the health impact of isolated oligohydramnios progressively decreases with gestational age, especially in the last trimester^81^. In fact in our study the rate of PCS among women affected by oligohydramnios was 23.8% (=10/42) in gestations <29 weeks, increased to 33.9% (=19/56) at 29–32 weeks and to 42.5% (=71/167) at 33–36 weeks, plummeting to 14.7% (=227/1,591) at 37–40 weeks and further declining to 8.7% (=65/748) at 41+ weeks. The respective UCS rates for the same gestation classes had a rather different trend, being 59.5% for gestations <29 weeks, 62.5% at 29–32 weeks, 38.3% at 33–36 weeks, 20.7% at 37–40 weeks and 23.9% at 41+ weeks.

The following important factors, predominantly associated with OCS than UCS, were maternal age ≥35 and pre-delivery LoS ≥ 3 days. Obesity, hypertension, diabetes and subsequent fetal anomalies are more likely in women older than 35^82^. Moreover, advanced maternal age increases the risk of spontaneous abortion, pre-term delivery, bleeding and stillbirth^83,84^. Nonetheless, the DM should be decided based upon the health condition of the parturient rather than just age^82^.

In the whole FVG region the adjusted rates over the years increased for UCS and UCS/OCS, not for OCS. A possible explanation for this interesting finding may be that the epidemiological profile of women delivering in FVG have changed over time, especially in terms of higher maternal age, since the proportion of parturients 40–44 years old progressively increased from 4.7% in 2005 to 6.0% in 2008, 6.7% in 2010, 7.9% in 2013 and 8.8% in 2015. However, misclassification issues shall not be ruled out.

Leaving aside placenta previa/abruptio placenta/ante-partum haemorrage, factors predominantly associated with UCS than OCS were PROM, post-term as well as pre-term gestations, labour analgesia and cord prolapse.

At any gestational age (pre-term or even at 42 weeks) the fetal membranes can rupture before labour onset. Since prolonged PROM increases the risk of ascending infections, labour (spontaneous or induced) is essential to accelerate the delivery and prevent potential perinatal deaths due to infections^85^. In our study the prevalence of PROM was 11.8% and labour was induced in 19.9% (=2,540/12,778) cases of PROM, of which 21.3% ended up with an OCS and 16.5% with UCS.

Since post-term gestations pose substantial health risk both for the woman and the child, labour can be quickly induced at 41 weeks or alternatively a CS may be considered^85^. According to some authors, a PCS at even 39 weeks may lower several potential untoward infant outcomes^86–89^. In the whole FVG the rate of labour induction among post-term gestations was 35.3% (=6,592/17,010), of which 23.1% (=15,24/6,592) ended up with an OCS. The latter figure is lower than 24.2%, the OCS rate in FVG during the whole study period.

Although CS is reportedly less likely among women undergoing labour induction than those managed expectantly^90–92^, induction seems implicated in the overuse of CS, due to clinical impatience in obstetric decision-making^74^. Labour mode was excluded from multivariable analysis in the present study (as explained in the methods). In FVG during the whole study period the crude rate of labour induction was 15.6% and labour was augmented (by oxytocin) in 6.2% cases, for a total 21.8% pharmacological perinatal interventions. In high-income countries the corresponding rates of labour induction are around 25% of all deliveries, continuously increasing^89^. In the UK and USA, labour is induced in about 20% deliveries^79,80,93^.

Labour analgesia was a further relevant factor more notably associated with UCS in our analysis, a finding previously reported by others^93^. Nonetheless, the impact of labour analgesia on the riks of UCS according to the open literature seems inconclusive, as endorsed by a Cochrane review on 20 RCT for a total 6,534 pregnancies^94^.

### Variability on DM between hospitals

The WHO recommends an evidence-based evaluation of health care services at the hospital level “*in a standardized and action oriented manner*, *with the inclusion of maternal and perinatal outcomes, in order to be able to provide adequate conclusions to format policies, practices and actions”*^8,95^.

All FVG hospitals showed a higher adjusted risk of OCS as compared with the reference (centre G) during 2005–2015. Out of these 10 hospitals with increased adjusted risk of OCS, 9 (A, B, C, D, E, F, I, J, K) performed less SVD and 5 (A, C, D, I, J) less IVD. In the latter five centres CS was therefore probably overused. Since our results are highly adjusted, the difference in hospital performance of DM we found in FVG during 2005–2015 presumably reflects variability in practice pattern.

Variability of DM by maternity centres has been reported also elsewehere^32^, and it was also found in the already mentioned prospective study conducted in FVG during 2006–2007 on 15,726 deliveries drawn from the same 11 regional centers^36^. The latter investigation reported higher crude risk estimates in all centers as compared with the referent, and in its fully adjusted model the number of hospitals showing higher CS rates was still 8^36^. Hospital variation on DM may be due to any of the following reasons: hospital efficiency and/or policy; practice pattern; unmeasured characteristics of the patients (such as maternal request of CS) residing in the corresponding hospital catchment area^26^.

Remarkable differences in CS rates are reported by healthcare sector in the open literature, with private facilities generally characterized by higher CS figures sustained by higher rates of maternal requests, hence economic interests are likely implied^96^. Explanatory factors as social/political pressures, maternal request, fear of medico-legal consequences, lack of systematic control/audits, poor attention of ward staff to performance efficiency and low level of care may have different impact on the CS rate in private versus public maternity facilites^25,34,35,97^. Likewise, the high CS rates in the entirety of Italy are seemingly influenced by CSs occurring in private health care facilities and in public hospitals with less than 1,000 annual deliveries^20,67^. However, the impact of the health care sector may vary by geograpical area, setting and type of health system. In the present study, the only private maternity unit of FVG (hospital C) showed the third lowest adjusted risk of OCS and the third lowest adjusted UCS risk in the entire region.

### Prospects

Although all in all maternity centres of FVG performed efficiently in terms of CS rate during 2005–2015 as compared with the rest of Italy, the hospital variation in DM rates we found could be targeted by policy interventions aimed at further reducing the recourse to unnecessary CS^25,33^. This might enable to bring the rate of OCS in FVG under 20%, close to figures of the Nordic countries^21^.

Various strategies (financial and non-financial) have been proposed to contain the CS rate in high income countries^32,35,98^. The optimal interventions should be multi-faceted and structured at the national, regional and local level, and be directed to users, providers and health systems^98,99^.

The elimination of economic incentives for CS and a revision of the medico-legal system are certainly critical steps^35,98^. There are suggestions in the literature of other possible financial strategies, such as penalties for institutions delivering undesirable care/health outcomes and economic rewards for those performing efficiently^33^.

Among non-financial strategies, labour companionship and midwife-led care have proven to increase the SVD rate, improve health outcomes, patient satisfaction and attain higher level of efficiency of health-care services^98^. Other relevant non-financial-policy interventions include updated training for health care staff, education of women, patient-centered care, improved communication between parturients and health professionals, informed decision making on DM and provision of adequate social/family support to the woman^33,98^.

From a clinical perspective, limiting primary CS, implementing VBAC, cephalic version for term deliveries with breech presentation in selected parturients and VD for vertex presenting twins may contribute to contain the recourse to CS^98^. In case of obstructed labour, CS should be averted before the establishment of an active phase (employing a 6 cm benchmark for cervix dilation), especially in nulliparas, labour induction and trial of labour after CS (TOLAC), pursuing IVD where feasible^65,74^. The inappropriate use of CS in nulliparas and RCS should be carefully monitored and subject to audit at hospital level^63^. Maternity centres should be enforced to follow evidence based standardized protocols recommended by the major international obstetric associations^34,35^.

Interventions aimed at reducing the use of CS should ideally be evaluated by randomized controlled trials, despite the difficulties in randomizing a woman during labour. Rigorous and systematic observational studies may be acceptable alternatives, preferably using comprehensive databases from health information systems as we did.

## Conclusions

The present study shows that routinely collected administrative data provide useful information for health planning and monitoring obstetric care. They are relatively inexpensive, do not require ad hoc collections, are accessible, annually updated, allowing for the assessment of time trends.

The OCS rate of 24.2% in FVG during 2005–2015 was importantly lower that the figure reported for the whole of Italy (38.1%) and slightly under the most recent average estimates from OECD countries, despite being higher than the benchmark recommended by WHO^5,20,92,98^. Although all in all maternity centres of FVG performed efficiently in terms of CS rates during 2005–2015 as compared with the rest of the country, we found high variability of DM across hospitals. Since a relevant proportion of CS in excess in high-income countries is considered unnecessary, the latter variability could be targeted by policy interventions aimed at further reducing the recourse to unnecessary CS, by enforcing adherence to standardized international obstetric guidelines at the hospital level.

Our study confirmed the main risk factors for CS according to the open literature. In some clinical conditions such as obstructed labor, breech presentation, non-reassuring fetal status, history of CS, higher maternal age, multiple birth and isolated oligohyramnions, consideration may be given to more conservative DM. The overuse of CS in primiparas and RCS should be carefully monitored and subject to audits. In case of obstructed labour, CS should be averted before the establishment of an active phase (employing a 6 cm benchmark for cervix dilation), especially in nulliparas, labour induction and TOLAC, attempting IVD where feasible.

The systematic methodology proposed in this study could be employed to monitor the rates of various DM in other geographical areas (including other Italian regions), influencing regional, national and (possibly) international health policies.

## Supplementary information

Supplementary File

## Data Availability

This study analysed third party data, extracted from the Regional Repository of Friuli Venezia Giulia (FVG), a database anonimously storing potentially sensitive information. Access to this database is therefore subject to permission from the Regional Health Authority of FVG. Contact: Epidemiology & Health Information Service; Central Health Directorate; Health & Social Integration; Social & Family Policies; Via Pozzuolo 330, 33100, Udine, Italy. Tel: +39 0432 805661; email: salute@certregione.fvg.it.
