# Author Correction: A Systematic Evaluation of Hospital Performance of Childbirth Delivery Modes and Associated Factors in the Friuli Venezia Giulia Region (North-Eastern Italy), 2005–2015

**DOI:** 10.1038/s41598-020-62742-0

**Published:** 2020-04-10

**Authors:** L. Cegolon, G. Mastrangelo, W. C. Heymann, G. Dal Pozzo, L. Ronfani, F. Barbone

**Affiliations:** 1 Local Health Unit N.2 “Marca Trevigiana”, Public Health Department, Treviso, Italy; 2 Institute for Maternal & Child Health, IRCCS “Burlo Garofolo”, Trieste, Italy; 3 Padua University, Department of Cardio-Thoracic & Vascular Sciences, Padua, Italy; 4 Florida State University, College of Medicine, Department of Clinical Sciences, Sarasota, Florida USA; 5 Florida Department of Health, Sarasota County Health Department, Sarasota, Florida USA; 6 Obstetrics & Gynecology Unit, Hospital “Villa Salus”, Venice, Italy

Correction to: *Scientific Reports* 10.1038/s41598-019-55389-z, published online 19 December 2019

The original version of this Article contained errors.

In the Abstract,

“Since improvement of obstetric care at the hospital level needs quantitative evidence, using routinely collected health data we contrasted the performance of the 11 maternity centres (coded with an alphabetic letter A to L) of an Italian region, Friuli Venezia Giulia (FVG), during 2005–15, after removing the effect of several factors associated with different delivery modes (DM): spontaneous vaginal delivery (SVD), instrumental vaginal delivery (IVD), overall CS (OCS) and urgent/emergency CS (UCS).”

now reads:

“Since improvement of obstetric care at the hospital level needs quantitative evidence, using routinely collected health data we contrasted the performance of the 11 maternity centres (coded with an alphabetic letter A to K) of an Italian region, Friuli Venezia Giulia (FVG), during 2005–15, after removing the effect of several factors associated with different delivery modes (DM): spontaneous vaginal delivery (SVD), instrumental vaginal delivery (IVD), overall CS (OCS) and urgent/emergency CS (UCS).”

“Although the overall CS rate in FVG during 2005–15 was 24.2%, well below the corresponding average Italian national figure (38.1%), the variability of DM rates across FVG maternity centres could be targeted by policy interventions aimed at reducing the recourse to unnecessary CS.”

now reads:

“Although the overall CS rate in FVG during 2005–15 was 24.2%, well below the corresponding average Italian national figure (38.1%), the variability of DM rates across FVG maternity centres could be targeted by policy interventions aimed at further reducing the recourse to unnecessary CS.”

Furthermore, in Figure 1 the number of vaginal deliveries was omitted. As a result the Figure legend,

“Flowchart displaying the criteria applied to the initial database to obtain the final number of hospital births available for the analysis. SVD  =  spontaneous vaginal deliveries; IVD  =  instrumental vaginal deliveries; CS  =  cesarean sections.”

now reads:

“Flowchart displaying the criteria applied to the initial database to obtain the final number of hospital births available for the analysis. VD = Vaginal Deliveries; SVD  =  spontaneous vaginal deliveries; IVD  =  instrumental vaginal deliveries; CS  =  cesarean sections; PCS: Planned CS; UCS = Urgent/Emergency CS”

In Figure 7 the number of Placenta previa/ abruptio placenta/ante-partum Hemorrage was incorrectly reported as 752. As a result, in the Methods section under subheading ‘Data Cleaning’,

“87 births by SVD and 15 births by IVD associated with placenta previa/abruptio placenta/ante-partum hemorrage have maintained their original DM, but recoded as not having any placenta previa/abruptio placenta/ante-partum hemorrhage.”

now reads:

“Placenta previa/abruptio placenta/ante-partum haemorrhage delivering by SVD (N = 136) and by IVD (N = 22) have been excluded from the analysis, for a total 158 missing data.”

Under the subheading ‘Conceptual Framework’,

“4. Obstetric conditions, shown in Fig. 7: oligohydramnios; polyhydramnios; eclampsia/pre-eclampsia; placenta previa/abruptio placenta/ante-partum hemorrhage; non- reassuring fetal status; fetal anomalies; cord prolapse; PROM; Rh immunization, obstructed labour (except shoulder girdle dystocia); labour analgesia; labour mode; presentation; any medical assisted fertilization.”

now reads:

“4. Obstetric conditions, shown in Fig. 7: oligohydramnios; polyhydramnios; eclampsia/pre-eclampsia; placenta previa/abruptio placenta/ante-partum hemorrhage; non- reassuring fetal status; Congenital malformations at birth; cord prolapse; PROM; Rh immunization, obstructed labour (except shoulder girdle dystocia); labour analgesia; labour mode; presentation; any medical assisted fertilization.”

Under the subheading ‘the database’,

“We used the following ICD-9 codes to retrieve the obstetric conditions associated with each childbirth:

Polyhydramnios: 657.0;

Oligohydramnios: 658.0;

Antepartum hemorrhage, abruptio placentae and placenta previa: 641.(0-1-2-3-8-9);

Obstructed labour (except shoulder girdle dystocia): 660.(0-1-2-3-5-6-7-8-9);

Non-reassuring fetal status: 656.3;

Fetal anomalies: 655.9;

Cord prolapse: 663.0;

Premature rupture of membranes (PROM): 658.1;

Eclampsia/pre-eclampsia: 624.(4-5-6-7);

Rh iso-immunization: 656.1.”

now reads:

“We used the following ICD-9 codes to retrieve the obstetric conditions associated with each childbirth:

Polyhydramnios: 657.0;

Oligohydramnios: 658.0;

Antepartum hemorrhage, abruptio placentae and placenta previa: 641.(0-1-2-3-8-9);

Obstructed labour (except shoulder girdle dystocia): 660.(0-1-2-3-5-6-7-8-9);

Non-reassuring fetal status: 656.3;

Cord prolapse: 663.0;

Premature rupture of membranes (PROM): 658.1;

Eclampsia/pre-eclampsia: 642.(4-5-6-7);

Rh iso-immunization: 656.1.”

Finally, the Supplementary File that originally accompanied this Article contained mathematical inaccuracies in the rounding of some estimates in Supplementary Tables S1 and S2.

These errors have now been corrected in the PDF and HTML versions of the Article and in the accompanying Supplementary Information file.

