# Supplementary material for: A Systematic Evaluation of Hospital Performance of Childbirth Delivery Modes and Associated Factors in the Friuli Venezia Giulia Region (North-Eastern Italy), 2005–2015
Source: Sci Rep. 2019 Dec 19;9:19442. doi: 10.1038/s41598-019-55389-z (PMC6923393; doi:10.1038/s41598-019-55389-z)
Supplement: Supplementary file 1 — Supplementary File [file 41598_2019_55389_MOESM1_ESM.pdf]

Supplementary Table 1. Multivariable logistic regression models contrasting each delivery mode (spontaneous vaginal delivery, SVD; instrumental vaginal delivery, IVD; overall caesarean sections, OCS; urgent/emergency cesarean sections, UCS) with the rest of births. The model for UCS/OCS contrasts the rate of UCS out of all OCS. Significant factors with the corresponding reference category: odds ratio (OR) with 95% confidence interval (in brackets) and Benjamini-Hochberg p-value estimated at 5% false discovery rate (bottom of each cell). Obs.= Complete case (analysis) observations. NA= not applicable; NS= non-significant. N=number. CS= cesarean section.

FACTORS	Reference category	DELIVERY MODE OR (95%CI) with BH p-value				
		SVD (104,566 obs.)	IVD (104,624 obs.)	OCS (104,567 obs.)	UCS (104,424 obs.)	UCS/OCS (24,582 obs.)
N. previous livebirth: 1	0	3.66 (3.50; 3.82)<br>0	0.24 (0.23; 0.27)<br>1.18E-263	0.42 (0.40; 0.44)<br>4.47E-265	0.45 (0.42; 0.47)<br>2.37E-161	0.80 (0.74; 0.87)<br>9.39E-07
N. previous livebirth: 2	0	6.88 (6.31; 7.51)<br>0	0.20 (0.17; 0.23)<br>6.14E-81	0.22 (0.20; 0.24)<br>9.57E-216	0.34 (0.31; 0.38)<br>1.89E-88	NS
N. previous CS: 1	0	0.04 (0.04; 0.04)<br>0	2.03 (1.79; 2.30)<br>8.18E-27	27.61(25.87; 29.47)<br>0	5.65 (5.25; 6.08)<br>0	0.54 (0.50; 0.60)<br>2.42E-37
Presentation: Breech	Cefalic	0.02 (0.02; 0.02)<br>0	0.09 (0.06; 0.14)<br>6.98E-33	85.10 (75.84; 95.48)<br>0	4.71 (4.37; 5.08)<br>0	0.49 (0.45; 0.53)<br>2.72E-63
Non reassuring fetal status: Yes	No	0.02 (0.02; 0.02)<br>0	4.30 (3.89; 4.76)<br>3.93E-179	11.86 (10.79; 13.04)<br>0	14.55 (13.27; 15.95)<br>0	6.68 (5.62 7.93)<br>1.36E-102
Obstructed labour (but shoulder girdle dystocia): Yes	No	NS	4.44 (4.06; 4.85)<br>2.65E-236	9.03 (8.29; 9.83)<br>0	11.42 (10.53; 12.39)<br>0	4.94 (4.26; 5.73)<br>1.87E-98
N. previous CS: 2	0	0.00 (0.00; 0.00)<br>6.08E-246	0.23 (0.08; 0.62)<br>0.0087	543.45 (386.46; 764.22)<br>5.36E-286	4.86 (4.03; 5.87)<br>2.49E-60	0.24 (0.20; 0.29)<br>6.02E-43
Placenta previa/ abruptio placenta/ante-partum hemorrage: Yes	No	NA	NA	NA	21.99 (18.83; 25.69)<br>0	2.21 (1.89; 2.58)<br>7.85E-23
N. previous livebirth: 3	0	8.82 (7.40; 10.51)<br>5.21E-129	0.15 (0.10; 0.22)<br>3.17E-21	0.18 (0.15; 0.21)<br>2.73E-73	0.30 (0.24; 0.36)<br>1.25E-31	NS
Mother's age: (40-44) years	20-24	0.30 (0.27; 0.34)<br>3.24E-112	1.51 (1.30; 1.76)<br>5.45E-07	3.34 (2.97; 3.75)<br>1.40E-89	2.03 (1.78; 2.31)<br>5.98E-26	0.81 (0.67; 0.97)<br>0.0404
Mutiple birth: twins or more	Singleton	0.14 (0.11; 0.16)<br>6.24E-97	NS	7.06 (5.86; 8.49)<br>3.29E-94	1.71 (1.47; 1.99)<br>6.89E-12	0.75 (0.64; 0.89)<br>0.0015
Placental weight: (600-999)g	500-599	0.68 (0.66; 0.71)<br>2.13E-80	NS	1.62 (1.54; 1.69)<br>9.38E-95	1.31 (1.24; 1.37)<br>1.01E-23	0.87 (0.81; 0.94)<br>0.0010
Mother's age: (35-39) years	20-24	0.46 (0.42; 0.50)<br>1.46E-75	1.27 (1.13; 1.43)<br>0.0002	2.24 (2.03; 2.47)<br>4.79E-59	1.68 (1.51; 1.87)<br>1.11E-20	NS
Eclampsia/pre-eclampsia: Yes	No	0.24 (0.21; 0.28)<br>2.97E-72	NS	4.87 (4.17; 5.68)<br>2.63E-89	4.73 (4.09; 5.48)<br>1.15E-94	2.16 (1.81; 2.58)<br>9.23E-17
N. previous livebirth: 4	0	14.42 (11.73; 19.40)<br>3.80E-69	0.05 (0.02; 0.14)<br>2.11E-08	0.12 (0.10; 0.17)<br>3.09E-40	0.26 (0.18; 0.36)<br>1.35E-15	NS
Placental weight: (1,000-1,500)g	500-599	0.22 (0.18; 0.27)<br>3.42E-50	0.58 (0.38; 0.90)<br>0.0295	5.94 (4.85; 7.29)<br>4.09E-65	1.64 (1.36; 1.98)<br>3.46E-07	0.70 (0.57; 0.86)<br>0.0012

Pre-delivery LoS: (3-5) years	< 3	0.51 (0.47; 0.56) 5.68E-48	0.80 (0.69; 0.93) 0.0100	2.35 (2.13; 2.58) 1.51E-67	1.30 (1.17; 1.45) 2.15E-06	0.56 (0.49; 0.64) 2.72E-16
Oligohydramnios: Yes	No	0.50 (0.45; 0.55) 5.11E-44	NS	2.53 (2.28; 2.81) 1.65E-68	2.21 (1.98; 2.48) 3.25E-43	NS
Mother's age: (30-34) years	20-24	0.58 (0.53; 0.62) 1.77E-44	1.17 (1.06; 1.31) 0.0081	1.75 (1.60; 1.92) 6.48E-34	1.46 (1.33; 1.62) 1.33E-13	NS
Mother's age: 45+ years	20-24	0.15 (0.11; 0.20) 2.10E-35	NS	8.13 (5.96; 11.08) 1.04E-39	2.96 (2.11; 4.16) 6.29E-10	NS
Birthweight: <2,000 g	2,500-4,000	0.30 (0.25; 0.36) 1.84E-34	0.30 (0.17; 0.52) 7.87E-05	4.29 (3.53; 5.21) 2.91E-48	3.17 (2.67; 3.75) 2.00E-39	1.62 (1.31; 1.99) 2.01E-05
Pre-delivery LoS: 6+ days	< 3	0.41 (0.36; 0.48) 7.86E-33	0.70 (0.51; 0.95) 0.0443	2.91 (2.51; 3.37) 9.21E-46	0.74 (0.64; 0.85) 7.69E-05	0.38 (0.33; 0.45) 3.94E-30
N. US scans during pregnancy: 6+	<4	0.76 (0.72; 0.80) 7.56E-23	NS	1.42 (1.33; 1.51) 5.08E-27	NS	0.70 (0.63; 0.78) 6.00E-11
Gestational age: 41+ weeks	37-40	0.82 (0.78; 0.85) 1.18E-19	1.25 (1.18; 1.33) 1.93E-12	1.07 (1.02; 1.13) 0.0082	1.51 (1.42; 1.59) 1.97E-44	1.98 (1.80; 2.18) 6.80E-42
Polyhydramnios: Yes	No	0.35 (0.28; 0.45) 1.13E-17	NS	3.34 (2.62; 4.25) 2.76E-22	2.89 (2.26; 3.69) 5.14E-17	NS
Gestational age: (33-36) weeks	37-40	0.69 (0.64; 0.75) 4.79E-17	0.68 (0.57; 0.80) 3.23E-05	1.75 (1.60; 1.92) 6.24E-34	2.11 (1.93; 2.30) 1.10E-59	2.51 (2.22; 2.83) 5.00E-49
Mother's educational level: Junior Secondary	University/higher	0.80 (0.76; 0.85) 9.73E-16	NS	1.28 (1.21; 1.36) 5.58E-17	1.19 (1.11; 1.27) 1.00E-06	NS
Mother's Nationality: Non-EU	Italian	0.80 (0.76; 0.85) 6.44E-15	1.24 (1.14; 1.35) 2.82E-06	1.25 (1.18; 1.33) 1.32E-12	1.29 (1.21; 1.38) 2.15E-13	1.24 (1.13; 1.36) 8.57E-06
Maternal age: (25-29) years	20-24	0.75 (0.69; 0.80) 6.47E-15	NS	1.38 (1.26; 1.50) 6.35E-13	1.24 (1.13; 1.36) 1.74E-05	NS
Birthweigth: (2,000-2,500) g	2,500-4,000	0.69 (0.63; 0.76) 4.46E-14	0.73 (0.61; 0.88) 0.0018	1.84 (1.66; 2.04) 2.14E-31	1.76 (1.59; 1.95) 5.92E-27	1.38 (1.21; 1.58) 6.49E-06
Cord prolapse: Yes	No	0.01 (0.00; 0.03) 5.13E-14	NS	117.80 (41.20; 336.77) 1.09E-18	159.33 (62.16; 408.37) 1.63E-25	54.69 (7.49; 399.25) 0.0002
Mother's educational level: Primary/None	University/higher	0.60 (0.52; 0.69) 7.11E-13	NS	1.61 (1.38; 1.87) 1.05E-09	1.41 (1.19; 1.66) 9.42E-05	NS
Labour analgesia: Yes	No	0.86 (0.82; 0.89) 8.98E-13	1.82 (1.71; 1.92) 1.77E-91	0.67 (0.63; 0.70) 3.05E-51	1.30 (1.23; 1.38) 9.01E-20	5.28 (4.67; 5.97) 3.40E-152
N. US scans during pregnancy: 4-5	<4	0.85 (0.80; 0.89) 1.42E-10	NS	1.23 (1.16; 1.30) 1.26E-11	NS	0.82 (0.74; 0.90) 0.0002
Birthweight: 4,000+ g	2,500-4,000	0.81 (0.76; 0.87) 1.35E-09	1.15 (1.04; 1.28) 0.0160	1.17 (1.08; 1.26) 9.20E-05	1.25 (1.15; 1.36) 4.44E-07	NS
Mother's occupation: Employed/Clerk	Unemployed/ student/housewife	1.17 (1.11; 1.23) 2.78E-09	NS	0.98 (0.97; 0.99) 2.37E-05	0.85 (0.79; 0.90) 1.10E-06	NS
Gestational age: <29 weeks	37-40	2.64 (1.91; 3.64) 6.11E-09	NS	0.45 (0.32; 0.62) 2.77E-06	2.29 (1.74; 3.01) 6.71E-09	8.50 (5.69; 12.69) 8.65E-25
Placental weight: <500g	500-599	1.15 (1.10; 1.21) 2.32E-08	NS	0.87 (0.82; 0.92) 1.94E-06	0.93 (0.87; 0.99) 0.0268	NS
Neonatal status: Stillborn	Liveborn	2.70 (1.88; 3.88) 1.09E-07	NS	0.31 (0.20; 0.47) 7.34E-08	0.35 (0.24; 0.50) 5.04E-08	NS

Hypertension/diabetes: Yes	No	0.74 (0.66; 0.83)<br>2.38E-07	NS	1.36 (1.21; 1.53)<br>8.68E-07	1.33 (1.18; 1.51)<br>1.15E-05	NS
Premature rupture of membranes (PROM): Yes	No	1.15 (1.09; 1.21)<br>3.11E-07	0.90 (0.83; 0.98)<br>0.0232	NS	1.93 (1.82; 2.05)<br>9.13E-103	4.87 (4.35; 5.44)<br>1.36E-168
Mother's educational level: Secondary	University/higher	0.91 (0.88; 0.95)<br>3.04E-05	NS	1.10 (1.05; 1.15)<br>6.56E-05	1.12 (1.07; 1.19)<br>2.62E-05	1.09 (1.01; 1.18)<br>0.0404
Rh iso-immunization: Yes	No	0.43 (0.26; 0.69)<br>0.0007	NS	2.84 (1.72; 4.69)<br>6.43E-05	NS	NS
Calendar year (2005-2015)	Linear term	NS	1.02 (1.01; 1.03)<br>0.0012	NS	1.01 (1.00; 1.02)<br>0.0032	1.04 (1.03; 1.05)<br>2.00E-13
Gestational age: (29-32) weeks	37-40	0.70 (0.54; 0.89)<br>0.0052	0.36 (0.16; 0.84)<br>0.0343	1.77 (1.38; 2.26)<br>1.03E-05	2.77 (2.24; 3.43)<br>1.21E-20	4.07 (3.10; 5.33)<br>1.76E-23
Mother's occupation: Employed (other)	Unemployed/<br>student/housewife	1.08 (1.02; 1.14)<br>0.0076	NS	NS	0.93 (0.87; 0.99)<br>0.0387	NS
Father's age: (15-19) years	30-34	1.92 (1.21; 3.03)<br>0.0076	NS	0.53 (0.30; 0.95)<br>0.0388	NS	NS
Number of previous intentional abortions: 1	0	1.09 (1.01; 1.16)<br>0.0252	0.79 (0.71; 0.89)<br>0.0002	NS	NS	NS
Number of previous spontaneous abortions: 1	0	0.94 (0.89; 0.99)<br>0.0313	NS	NS	NS	NS
Number of obstetric checks in pregnancy: 8+	4-7	0.95 (0.91; 1.00)<br>0.0397	NS	1.08 (1.03; 1.14)<br>0.0046	1.06 (1.01; 1.13)<br>0.0368	NS
Mother's age: (15-19) years	20-24	1.21 (1.01; 1.45)<br>0.0491	NS	NS	NS	NS
Number of previous intentional abortions : 2+	0	NS	NS	1.25 (1.07; 1.47)<br>0.0071	1.25 (1.05; 1.48)<br>0.0150	NS
Number of obstetric checks in pregnancy: <4	4-7	NS	NS	1.06 (1.01; 1.11)<br>0.0311	0.94 (0.88; 1.00)<br>0.0431	0.85 (0.78; 0.92)<br>0.0002
Father's age: 55+ years	30-34	NS	NS	1.31 (1.03; 1.67)<br>0.0311	NS	NS
Any medical assisted fertilization: Yes	No	NS	NS	1.26 (1.01; 1.56)<br>0.0416	0.68 (0.55; 0.85)<br>0.0010	0.65 (0.50; 0.83)<br>0.0013

Results of Supplementary Tables 1 and 2 are outputs from the same multivariable logistic models, adjusted for all the following factors:

SVD:

- **Health care setting and timeframe:** hospital;
- **Maternal health factors**: maternal age; hypertension/diabetes; number of obstetric checks in pregnancy; number of US scans in pregnancy; pre-delivery LoS; neonatal status;
- **Child's fragility factors:** multiple birth;
- **Child's size factors:** gestational age; birthweight; placental weight;
- **Obstetric history factors:** number of previous livebirths; number of previous CS; number of previous spontaneous abortions; number of previous intentional abortions;
- **Socio-demographic factors**: father's age; mother's nationality; mother's education; mother's occupation;
- **Obstetric factors**: eclampsia/pre-eclampsia; polyhydramnios; oligohydramnios; non-reassuring foetal status; PROM; presentation; obstructed labour; labour analgesia; cord prolapse; Rh iso-immunization

IVD:

- **Health care setting and timeframe:** hospital; calendar year;
- **Maternal health factors**: maternal age; pre-delivery LoS;
- **Child's fragility factors:** multiple birth;
- **Child's size factors:** gestational age; birthweight; placental weight;
- **Obstetric history factors:** number of previous livebirths; number of previous CS; number of previous spontaneous abortions; number of previous intentional abortions
- **Socio-demographic factors**: father's age; mother's nationality; mother's occupational status;
- **Obstetric factors**: non-reassuring foetal status; presentation; PROM; obstructed labour; labour analgesia.

OCS:

- **Health care setting and timeframe:** hospital;
  Maternal health factors: maternal age; hypertension/diabetes; number of obstetric checks in pregnancy; number of US scans in pregnancy; neonatal status; pre-delivery LoS; any medical assisted fertilization;
- **Child's fragility factors:** multiple birth;
- **Child's size factors:** gestational age; birthweight; placenta weight;
- **Obstetric history factors:** number of previous livebirths; number of previous CS; number of previous intentional abortions;
- **Socio-demographic factors:** father's age; mother's nationality; mother's educational level; mother's occupational status;
- **Obstetric factors**: eclampsia/pre-eclampsia; polyhydramnios; oligohydramnios; non-reassuring foetal status; presentation; labour analgesia; obstructed labour; cord prolapse; Rh iso-immunization

UCS

- **Health care setting and timeframe:** hospital; calendar year;
- **Maternal health factors**: maternal age; number of obstetric checks in pregnancy; pre-delivery LoS; neonatal status;
  Child's size factors: gestational age; birthweight; placental weight;
- **Child's fragility factors:** multiple birth;
- **Obstetric history factors:** number of previous livebirths; number of previous CS; number of previous intentional abortions;
- **Socio-demographic factors**: fathers' age; mother's nationality; mother's education; mother's occupational status;
- **Obstetric factors**: eclampsia/pre-eclampsia; polyhydramnios; oligohydramnios; non-reassuring foetal status; presentation; placenta previa/abruptio placenta/antepartum hemorrage; PROM; obstructed labour; labour analgesia; cord prolapse; any medical assisted fertilization.

<u>UCS/OCS</u>

- **Health care setting and timeframe:** hospital; calendar year;
- **Maternal health factors**: mother's age; number of obstetric checks in pregnancy; number of US scans in pregnancy; pre-delivery LoS; neonatal status;
- **Child's size factors:** gestational age; birthweight; placental weight;
- **Child's fragility factors:** multiple birth;
- **Obstetric history factors:** number of previous livebirths; number of previous CS; number of previous spontaneous abortions; number of previous stillbirths;
- **Socio-demographic factors**: father's age; mother's nationality; mother's education;
- **Obstetric factors**: eclampsia/pre-eclampsia; polyhydramnios; non-reassuring foetal status; presentation; placenta previa/abruptio placenta/antepartum hemorrage; PROM; obstructed labour; labour analgesia; cord prolapse; any medical assisted fertilization.

Supplementary Table 2. Multivariable logistic regression models contrasting each delivery mode (spontaneous vaginal delivery, SVD; instrumental vaginal delivery, IVD; overall caesarean section, OCS; urgent/emergency cesarean sections, UCS) with the rest of births. The model for UCS/OCS contrasts the rate of UCS out of all OCS. Hospital estimates (odds ratio, OR) with 95% confidence interval (in brackets) and Benjamini-Hochberg p-value, estimated at 5% false discovery rate (bottom of each cell). NS= non-significant. Obs.= complete case (analysis) observations.

HOSPITAL	DELIVERY MODE OR (95%CI); with BH p-value				
	**SVD** (104,566 obs.)	**IVD** (104,624 obs.)	**OCS** (104,567 obs.)	**UCS** (104,424 obs.)	**UCS/OCS** (24,582 obs.)
**A**	0.70 (0.65; 0.76) 1.73E-18	0.80 (0.72; 0.88) 5.21E-05	1.94 (1.76; 2.12) 9.92E-44	1.48 (1.33; 1.63) 1.17E-13	0.76 (0.65; 0.89) 0.0010
**B**	0.39 (0.36; 0.42) 1.83E-128	NS	3.52 (3.22; 3.86) 3.35E-159	1.97 (1.78; 2.17) 6.15E-40	0.65 (0.56; 0.76) 1.16E-07
**C**	0.83 (0.76; 0.90) 3.87E-05	0.53 (0.46; 0.61) 1.44E-18	1.56 (1.41; 1.74) 2.35E-16	1.29 (1.15; 1.46) 3.02E-05	NS
**D**	0.47 (0.42; 0.53) 1.13E-38	0.44 (0.35; 0.54) 1.65E-13	3.08 (2.71; 3.50) 2.92E-65	2.99 (2.60; 3.43) 4.14E-54	1.51 (1.23; 1.86) 0.0002
**E**	0.39 (0.36; 0.43) 1.99E-85	NS	3.36 (3.01; 3.74) 7.44E-104	1.83 (1.62; 2.08) 9.36E-21	0.59 (0.50; 0.71) 7.16E-08
**F**	0.49 (0.45; 0.54) 1.33E-48	1.32 (1.17; 1.50) 4.79E-05	2.08 (1.86; 2.33) 1.87E-37	1.79 (1.58; 2.02) 5.11E-20	NS
**G**	reference	reference	reference	reference	reference
**H**	NS	NS	1.31 (1.19; 1.46) 2.80E-07	1.37 (1.23; 1.53) 4.22E-08	NS
**I**	0.53 (0.48; 0.58) 2.54E-38	0.84 (0.73; 0.96) 0.0244	2.56 (2.29; 2.87) 4.36E-59	1.74 (1.53; 1.97) 1.14E-17	0.73 (0.61; 0.88) 0.0022
**J**	0.58 (0.53; 0.63) 6.95E-39	0.79 (0.71; 0.89) 0.0001	2.24 (2.03; 2.46) 3.88E-60	1.63 (1.47; 1.81) 1.74E-19	0.79 (0.68; 0.93) 0.0080
**K**	0.59 (0.54; 0.65) 1.19E-30	NS	1.84 (1.65; 2.05) 1.17E-28	NS	0.55 (0.46; 0.65) 7.93E-11

Results of Supplementary Tables 1 and 2 are outputs from the same multivariable logistic models, adjusted for all the following factors:

SVD:

- **Health care setting and timeframe:** hospital;
- **Maternal health factors**: maternal age; hypertension/diabetes; number of obstetric checks in pregnancy; number of US scans in pregnancy; pre-delivery LoS; neonatal status;
- **Child's fragility factors:** multiple birth;
- **Child's size factors:** gestational age; birthweight; placental weight;
- **Obstetric history factors:** number of previous livebirths; number of previous CS; number of previous spontaneous abortions; number of previous intentional abortions;
- **Socio-demographic factors**: father's age; mother's nationality; mother's education; mother's occupation;
- **Obstetric factors**: eclampsia/pre-eclampsia; polyhydramnios; oligohydramnios; non-reassuring foetal status; PROM; presentation; obstructed labour; labour analgesia; cord prolapse; Rh-iso-immunization.

IVD:

- **Health care setting and timeframe:** hospital; calendar year;
- **Maternal health factors**: maternal age; pre-delivery LoS; fetoscopy; number of obestric checks in pregnancy;
- **Child's fragility factors:** Multiple birth;
- **Child's size factors:** gestational age; birthweight; placental weight;
- **Obstetric history factors:** number of previous livebirths; number of previous CS; number of previous spontaneous abortions; number of previous intentional abortions;
- **Socio-demographic factors**: father's age; mother's nationality; mother's occupational status;
- **Obstetric factors**: non-reassuring foetal status; presentation; PROM; obstructed labour; labour analgesia.

OCS:

- **Health care setting and timeframe:** hospital;
  Maternal health factors: maternal age; hypertension/diabetes; number of obstetric checks in pregnancy; number of US scans in pregnancy; neonatal status; pre-delivery LoS; any medical assisted fertilization;
- **Child's fragility factors:** multiple birth;
- **Child's size factors:** gestational age; birthweight; placenta weight;
- **Obstetric history factors:** number of previous livebirths; number of previous CS; number of previous intentional abortions;
- **Socio-demographic factors:** father's age; mother's nationality; mother's educational level; mother's occupational status;

- **Obstetric factors**: eclampsia/pre-eclampsia; polyhydramnios; oligohydramnios; non-reassuring fetal status; presentation; labour analgesia; obstructed labour; cord prolapse; Rh iso-immunization.

<u>**UCS**</u>

- **Health care setting and timeframe:** hospital; calendar year;
- **Maternal health factors**: maternal age; number of obstetric checks in pregnancy; pre-delivery LoS; neonatal status;
  Child's size factors: gestational age; birthweight; placental weight;
- **Child's fragility factors:** multiple birth;
- **Obstetric history factors:** number of previous livebirths; number of previous CS; number of previous intentional abortions;
- **Socio-demographic factors**: fathers' age; mother's nationality; mother's education; mother's occupational status;
- **Obstetric factors**: eclampsia/pre-eclampsia; polyhydramnios; oligohydramnios; non-reassuring foetal status; presentation; placenta previa/abruptio placenta/antepartum hemorrage; PROM; obstructed labour; labour analgesia; cord prolapse; any medical assisted fertilization.

<u>**UCS/OCS**</u>

- **Health care setting and timeframe:** hospital; calendar year;
- **Maternal health factors**: mother's age; number of obstetric checks in pregnancy; number of US scans in pregnancy; pre-delivery LoS; neonatal status;
- **Child's size factors:** gestational age; birthweight; placental weight;
- **Child's fragility factors:** multiple birth;
- **Obstetric history factors:** number of previous livebirths; number of previous CS; number of previous spontaneous abortions; number of previous stillbirths;
- **Socio-demographic factors**: father's age; mother's nationality; mother's education;
- **Obstetric factors**: eclampsia/pre-eclampsia; polyhydramnios; non-reassuring foetal status; presentation; placenta previa/abruptio placenta/antepartum hemorrage; PROM; obstructed labour; labour analgesia; cord prolapse; any medical assisted fertilization.

CERTIFICATO DI ASSISTENZA AL PARTO

Timbro del Reparto

Io sottoscritto/a ______________________ C.R.A. (1) ______________

in qualità di: 1 ☐ medico ostetrico/a 2 ☐ ostetrico/a 3 ☐ altro

certifico quanto segue:

A) INFORMAZIONI SOCIO-DEMOGRAFICHE SULLA MADRE:

cognome da nubile (2) ______________ nome ______________

nata a (2) ______________ pv (2) ____ data di nascita (2) ________ C.R.A. (2) ________

residente a (2) ______________ pv ____ cittadinanza ______________

condizione professionale/non professionale (barrare una sola voce):

- 1 ☐ occupata
- 2 ☐ disoccupata
- 3 ☐ in cerca di prima occupazione
- 4 ☐ studente
- 5 ☐ casalinga
- 6 ☐ altra condizione

posizione nella professione (se **occupata** barrare una sola voce):

- 1 ☐ imprenditore o libero professionista
- 2 ☐ altro lavoratore autonomo
- 3 ☐ lav. dip.: dirigente/direttivo
- 4 ☐ lav. dip.: impiegato
- 5 ☐ lav. dip.: operaio
- 6 ☐ altro lav. dip.

ramo di attività (se **occupata** barrare una sola voce):

- 1 ☐ agricoltura, caccia, pesca
- 2 ☐ industria
- 3 ☐ comm., pubbl. eserc., alberghi
- 4 ☐ pubblica amministrazione
- 5 ☐ altri servizi privati

titolo di studio conseguito (barrare una sola voce):

- 1 ☐ laurea
- 2 ☐ diploma univ. o laurea breve
- 3 ☐ diploma di scuola media superiore
- 4 ☐ diploma di scuola media inferiore
- 5 ☐ licenza elementare/nessun titolo

stato civile (barrare una sola voce):

- 1 ☐ non coniugata
- 2 ☐ coniugata
- 3 ☐ separata
- 4 ☐ vedova
- 5 ☐ divorziata
- 6 ☐ convivente

data di matrimonio (per coniugata/vedova): ______________

cognome/nome coniuge ______________ nato il ________ C.R.A. (1) ________

INFORMAZIONI SOCIO-DEMOGRAFICHE SUL PADRE (3):

cognome ______________ nome ______________

nato a ______________ pv ____ data di nascita ________ C.R.A. (1) ________

residente a ______________ pv ____ cittadinanza ______________

condizione professionale/non professionale (barrare una sola voce):

- 1 ☐ occupato
- 2 ☐ disoccupato
- 3 ☐ in cerca di prima occupazione
- 4 ☐ studente
- 5 ☐ casalingo
- 6 ☐ altra condizione

posizione nella professione (se **occupato** barrare una sola voce):

- 1 ☐ imprenditore o libero professionista
- 2 ☐ altro lavoratore autonomo
- 3 ☐ lav. dip.: dirigente/direttivo
- 4 ☐ lav. dip.: impiegato
- 5 ☐ lav. dip.: operaio
- 6 ☐ altro lav. dip.

ramo di attività (se **occupato** barrare una sola voce):

- 1 ☐ agricoltura, caccia, pesca
- 2 ☐ industria
- 3 ☐ comm., pubbl. eserc., alberghi
- 4 ☐ pubblica amministrazione
- 5 ☐ altri servizi privati

titolo di studio conseguito (barrare una sola voce):

- 1 ☐ laurea
- 2 ☐ diploma univ. o laurea breve
- 3 ☐ diploma di scuola media superiore
- 4 ☐ diploma di scuola media inferiore
- 5 ☐ licenza elementare/nessun titolo

Mod. SAN. [illegible] - IMOCO s.p.a. - Villorba (TV) - Tel. 0422.9141

INFORMAZIONI RELATIVE AD EVENTUALI GRAVIDANZE PRECEDENTI:

numero parti precedenti: ___________________ numero delle I.V.G.:___________________________

numero totale dei nati vivi (4): _______________ figli deceduti nel primo mese di vita (8): ___________

numero dei nati morti (5): ___________________ numero dei cesarei precedenti: _________________

numero dei nati vivi pret. (<37s.) (6): ___________ anno ultimo parto: ___________________________

numero degli aborti spontanei (7): ____________

B) INFORMAZIONI RELATIVE ALL'ATTUALE GRAVIDANZA:

numero di visite di controllo in gravidanza (n.): ______

prima visita di controllo in gravidanza (sett.): _______

numero di ecografie in gravidanza: _______

indagini prenatali (barrare tutte le voci):

amniocentesi	1a ☐ si	1b ☐ no
prelievo villi coriali	2a ☐ si	2b ☐ no
fetoscopia/funicolocentesi	3a ☐ si	3b ☐ no
ecografia dopo la 22.esima sett.	4a ☐ si	4b ☐ no
decorso della gravidanza	1 ☐ fisiologico	2 ☐ patologico
ricoveri in ostetricia durante la gravidanza	1 ☐ si	2 ☐ no
ipertensione arteriosa in gravidanza trattata con farmaci	1 ☐ si	2 ☐ no
difetto di accrescimento fetale (9)	1 ☐ si	2 ☐ no

concepimento con tecniche di procreazione medico-assistita (se **sì** barrare una sola voce) (10):

1 ☐ solo trattamento farmacologico per induzione dell'ovulazione
2 ☐ IUI trasferimento di gameti maschili nella cavità uterina
3 ☐ GIFT trasferimento di gameti nelle tube di Falloppio
4 ☐ FIVET fecondazione in vitro e trasferimento degli embrioni in utero
5 ☐ ICSI fecondazione in vitro tramite iniezione di spermatozoi nell'ovocita e trasferimento degli embrioni in utero
6 ☐ altre tecniche

C1) INFORMAZIONI SUL PARTO:

data ____________________ ora _______________

luogo del parto (11):

1 ☐ istituto pubblico/privato 3 ☐ altra struttura
2 ☐ abitazione 4 ☐ altro

codice istituto__________________ denominazione ____

comune ________________________________ via ________________________________ n° ______

personale sanitario presente al parto al momento della nascita (barrare una o più voci):

1 ☐ ostetrico/a 4 ☐ anestesista
2 ☐ ostetrico-ginecologo 5 ☐ altro personale sanitario o tecnico
3 ☐ pediatra/neonatologo

altre presenze in sala parto (barrare una o più voci):

1 ☐ padre 2 ☐ persona di famiglia 3 ☐ persona di fiducia

numero di figli nati dal parto: maschi: _______ femmine:______

sett. di amenorrea prima del parto: ________

modalità del travaglio (barrare una sola voce) (12):

1 ☐ spontaneo 2 ☐ indotto con farmaci 3 ☐ pilotato 4 ☐ senza travaglio

modalità del parto (barrare una sola voce) (13):

1 ☐ spontaneo (vaginale senza ventosa o forcipe) 4 ☐ forcipe
2 ☐ cesareo in elezione o per fallita induzione 5 ☐ ventosa
3 ☐ cesareo in travaglio o in urgenza 6 ☐ altre modalità di parto per via vaginale

secondamento: 1 ☐ spontaneo 2 ☐ manuale e/o strumentale

peso della placenta:______________ (gr.)

analgesia praticata durante il travaglio: 1 ☐ si 2 ☐ no

profilassi RH: 1 ☐ si 2 ☐ no

consanguineità tra madre e padre (se **sì** barrare una sola voce):

1 ☐ parenti 4° gr. (figli di fratelli/sorelle)
2 ☐ parenti 5° gr. (coniuge sposato con figlio/a di un suo primo cugino)
3 ☐ parenti 6° gr. (secondi cugini)

C2) INFORMAZIONI SUL NEONATO (14):

cognome ________________ nome ________________ sesso_____

data di nascita: ______________ ora: ________

presentazione al parto (barrare una sola voce):

1 ☐ vertice
5 ☐ fronte
2 ☐ podice
6 ☐ bregma
3 ☐ spalla
7 ☐ altra
4 ☐ faccia

necessità di rianimazione (15): 1 ☐ si 2 ☐ no

se **sì** indicare il personale sanitario che ha avviato la rianimazione nei primi 5 minuti (barrare una sola voce):

1 ☐ ostetrico/a
5 ☐ anestesista-rianimatore
2 ☐ medico ostetrico
6 ☐ altro medico
3 ☐ pediatra
7 ☐ altro ruolo profess. sanit.
4 ☐ neonatologo/a
8 ☐ altra persona

punteggi APGAR rilevati al: primo minuto: ______ quinto minuto: _______

vitalità: 1 ☐ nato vivo (4) 2 ☐ nato morto **(compilare la sezione D)** (5)

necessità di trasferimento in terapia intensiva neonatale: 1 ☐ si 2 ☐ no

peso: __________ (gr.) lunghezza: __________ (cm) circ. cranica: ________ (mm)

tipo dei genitali esterni (16): 1 ☐ maschile 2 ☐ femminile 3 ☐ incerto

presenza di malformazioni (17): 1 ☐ assenti 2 ☐ presenti **(compilare la sezione E)**

data di compilazione ____________ ora __________

NOTE PER LA COMPILAZIONE DEL CERTIFICATO DI ASSISTENZA AL PARTO

1. Il CRA del personale sanitario che compila il certificato e del padre, deve essere considerato facoltativo ed utile alla sola registrazione informatizzata dei dati.
2. In caso di donna che vuole partorire in anonimato (figlio non riconosciuto o di filiazione ignota) "DONNA CHE NON VUOLE ESSERE NOMINATA" indicare il codice 999 per Cognome e Nome; non devono essere indicati il Codice sanitario, i comuni di nascita e di residenza; per la provincia di residenza indicare codice 999; indicare per esteso la provincia di nascita e solo l'anno per la data di nascita.
3. Nel caso di padre che non vuole essere nominato indicare codice 999 come nel caso di "DONNA CHE NON VUOLE ESSERE NOMINATA".
4. Per nato vivo si intende il prodotto del concepimento che, al momento della nascita, respiri o dimostri qualsiasi altro segno di vita quali, il battito cardiaco o la pulsazione del cordone ombelicale o movimenti dei muscoli volontari, indipendentemente dalla durata della gestazione
5. Per nato morto si intende il prodotto del concepimento che, una volta espulso o completamente estratto dal corpo materno, non abbia respirato o manifestato alcun segno di vita (battito cardiaco o la pulsazione del cordone ombelicale o movimenti dei muscoli volontari), purché siano trascorsi almeno 180 giorni di amenorrea. NEL CASO DI NATO MORTO DEVE ESSERE COMPILATA LA SEZIONE D) DEL CEDAP; NON DEVE ESSERE COMPILATA LA SCHEDA DI MORTE ENTRO IL PRIMO ANNO DI VITA.
6. Per nato pretermine si intende qualsiasi nato vivo con meno di 37 settimane complete di età di gestazione (cioè nato entro il 259° giorno compiuto), calcolata a partire dal primo giorno dell'ultimo ciclo mestruale.
7. Per aborto spontaneo si intende il prodotto del concepimento che, una volta espulso o completamente estratto dal corpo materno, non abbia respirato o manifestato alcun segno di vita (battito cardiaco o la pulsazione del cordone ombelicale o movimenti dei muscoli volontari), purché l'evento si verifichi entro il 180° giorno di amenorrea.
8. Morto nel primo mese di vita (morte neonatale secondo la legge italiana) è un soggetto nato vivo (vedere nota 4) e deceduto entro il compimento del 30° giorno di vita dal momento della nascita.

   Nel caso di morte neonatale e in generale nel caso di morte entro il compimento del 1° anno di vita (età inferiore a 365 giorni) deve essere compilata dal medico la SCHEDA DI MORTE ENTRO IL PRIMO ANNO DI VITA (mod. ISTAT/D4 e ISTAT/D5).
9. Per difetto di accrescimento fetale si intende il rallentato accrescimento intrauterino (valori inferiori al 10° percentile) diagnosticato in fase prenatale.
10. Nel caso il concepimento sia avvenuto attraverso l'utilizzo di tecniche di riproduzione medico-assistita specificare il metodo seguito:
    1 - solo trattamento farmacologico per induzione dell'ovulazione;
    2 - IUI (Intra Uterine Insemination);
    3 - GIFT (Gamete Intra Fallopian Transfer);
    4 - FIVET (Fertilization in Vitro and Embryo Transfer);
    5 - ICSI (Intra Cytoplasmic Sperm Injection);
    6 - altre tecniche.
11. "Luogo del parto": se avvenuto in "istituto pubblico-privato" (1) oppure in "altra struttura" (3) indicare il codice dell'istituto e la denominazione; se avvenuto in "abitazione" (2) o in "altro" (4) indicare il comune e l'indirizzo.
12. 'Modalità del travaglio': per induzione del travaglio con farmaci si intende l'utilizzo di ossitocina, prostaglandine. Per travaglio pilotato si intende l'uso di farmaci durante il travaglio, magari insorto spontaneamente, per correggerne le devianze dalla normalità. Con la modalità "senza travaglio" si identificano i casi in cui si procede in elezione o d'urgenza ad un Taglio Cesareo senza che si sia avviato spontaneamente o sia stato indotto un travaglio di parto.
13. 'Modalità del parto': con "parto spontaneo" si delimita il concetto di spontaneità all'assenza di operatività vaginale; viceversa devono essere inclusi i parti in cui si pratichi l'episiotomia. Con 'cesareo in elezione' devono essere considerati anche i casi conseguenti ad una fallita induzione, che non sono una vera e propria elezione, ma sono prossimi a questa. Con 'cesareo in travaglio' devono essere considerati anche i casi di Taglio Cesareo in urgenza (es. placenta previa, distacco di placenta). Il parto cesareo deve essere registrato solo con i codici 2) cesareo in elezione o per fallita induzione e 3) cesareo in travaglio o in urgenza. Il ricorso al cesareo, conseguente a una richiesta della donna, valutata e accettata dal clinico deve essere registrata con codice 2).

    Nel caso di parti plurimi la variabile 'Modalità del parto' può venire registrata per ogni singolo nato.
14. Nel caso di parto plurimo le notizie riguardanti i nati successivi al primo devono essere riportate nel MODULO AGGIUNTIVO PER I PARTI PLURIMI.
15. Non debbono essere ritenute "rianimazione" le comuni stimolazioni fisiche (le piccole percussioni del torace, sulle piante dei piedi, etc., la detersione del volto, etc.) le modeste supplementazioni di ossigeno somministrate per pochi secondi e, in generale, le manovre che rientrano nell'ambito delle cure minime riservate ai neonati con Apgar al primo minuto normale o lievemente inferiore a 7, ma con pronta ripresa e successiva gestione sovrapponibile a quella riservata ai neonati considerati senza problemi.
16. L'indicazione di sesso incerto ha valore ai fini conoscitivi e non altera le norme vigenti relative all'obbligo di dichiarazione del sesso alla nascita, né influisce sulla successiva eventuale modifica del sesso.
17. In presenza di nati vivi con malformazioni congenite viene compilata, da parte del medico accertatore, la sezione E del certificato, che sostituisce il "modello 51 sanità pubblica", concernente la denuncia di nato con malformazioni congenite, quale strumento di base utile per la rilevazione dei dati essenziali (art. 3 del Decreto 16 luglio 2001, n. 349).

D) INFORMAZIONI SULLE CAUSE DI NATI-MORTALITÀ (codici ICD-9-CM) (5):

malattia o condizione morbosa principale del feto ______________________ :__________

____

altra malattia o condizione morbosa del feto______________________ :__________

____

malattia o condizione morbosa principale della madre interessante il feto ______________ :__________

____

altra malattia o condizione morbosa della madre interessante il feto ______________ :__________

____

altra circostanza rilevante ______________________ :__________

____

momento della morte (barrare una sola voce):

1 ☐ prima del travaglio
3 ☐ durante il parto
2 ☐ durante il travaglio
4 ☐ sconosciuto

esecuzione esami strumentali in caso di malformazioni:

1 ☐ si
2 ☐ no

esecuzione fotografie in caso di malformazioni:

1 ☐ si
2 ☐ no

riscontro autoptico:

1 ☐ causa della morte confermata dall'autopsia
2 ☐ risultato dell'autopsia non ancora disponibile
3 ☐ autopsia non effettuata

E) INFORMAZIONI SULLA PRESENZA DI MALFORMAZIONI (solo per i nati vivi):

malformazioni diagnosticate (indicare massimo 3 malformazioni - codici ICD-9-CM):

1a m. ______________________ :__________
2a m. ______________________ :__________
3a m. ______________________ :__________

cariotipo del nato (se effettuato prima della nascita):

____

età gestazionale alla diagnosi di malformazione (in settimane compiute): _________ 99 ☐ Non rilevato

età neonatale alla diagnosi di malformazione (in giorni compiuti): _________ 99 ☐ Non rilevato

eventuali malformazioni in famiglia (barrare una o più voci):

fratelli	1 ☐ si	2 ☐ no
madre	1 ☐ si	2 ☐ no
padre	1 ☐ si	2 ☐ no
genitori madre	1 ☐ si	2 ☐ no
genitori padre	1 ☐ si	2 ☐ no
altri parenti madre (fratelli/cugini/zii)	1 ☐ si	2 ☐ no
altri parenti padre (fratelli/cugini/zii)	1 ☐ si	2 ☐ no

malattie insorte in gravidanza (indicare massimo 2 malattie - codici ICD-9-CM):

1a m. ______________________ :__________
2a m. ______________________ :__________

data di compilazione ______________

FIRMA DEL MEDICO

____
